# Long Noncoding RNA *HITTERS* Protects Oral Squamous Cell Carcinoma Cells from Endoplasmic Reticulum Stress‐Induced Apoptosis via Promoting MRE11‐RAD50‐NBS1 Complex Formation

**DOI:** 10.1002/advs.202002747

**Published:** 2020-10-04

**Authors:** Chenzhou Wu, Wen Chen, Fanyuan Yu, Yihang Yuan, Yafei Chen, Douglas R. Hurst, Yi Li, Longjiang Li, Zhe Liu

**Affiliations:** ^1^ State Key Laboratory of Oral Diseases and National Clinical Research Center for Oral Diseases and Department of Head and Neck Oncology West China Hospital of Stomatology Sichuan University Number 14, Unit 3, Renmin Nan Road Chengdu Sichuan 610041 China; ^2^ State Key Laboratory of Oral Diseases and National Clinical Research Center for Oral Diseases and Department of Endodontics West China Hospital of Stomatology Sichuan University Number 14, Unit 3, Renmin Nan Road Chengdu Sichuan 610041 China; ^3^ Department of Pathology University of Alabama at Birmingham Birmingham AL 35294 USA

**Keywords:** apoptosis, DNA damage response, endoplasmic reticulum stress, long noncoding RNA, oral squamous cell carcinoma

## Abstract

Recent studies have proven that long noncoding RNAs (lncRNAs) exhibit regulatory functions of both DNA damage response (DDR) and endoplasmic reticulum (ER) stress. Herein, ER stress‐induced lncRNA transcriptomic changes are reported in human oral squamous cell carcinoma (OSCC) cells and a novel lncRNA *HITTERS* (***H*** *ERPUD1* **i**n**t**ronic **t**ranscript of **ER s**tress) is identified as the most significantly upregulated lncRNA. It is shown that *HITTERS* is a nucleus‐located lncRNA including two transcript variants. *HITTERS* lacks an independent promoter but shares the same promoter with *HERPUD1*. *HITTERS* is transcriptionally regulated by *Activating Transcription Factor (ATF) 6*, *ATF4*, *X‐Box Binding Protein 1 (XBP1)*, and DNA methylation. In human OSCC tissues, *HITTERS* is significantly correlated with OSCC clinicopathological features and prognosis. Gain‐ and loss‐of‐function studies reveal that *HITTERS* promotes OSCC proliferation and invasion via influencing the expression of growth factor receptors and the downstream pathways. Once ER stress is triggered, *HITTERS* significantly attenuates ER stress‐induced apoptosis both in vivo and in vitro. Mechanically, *HITTERS* functions as RNA scaffold to promote MRE11‐RAD50‐NBS1 complex formation in the repair of ER stress‐induced DNA damage. To sum up, this study presents a novel lncRNA, namely *HITTERS*, which links ER stress and DDR together in OSCC.

## Graphical abstract

*HERPUD1* intronic transcript of endoplasmic reticulum (ER) stress (*HITTERS*) is a long noncoding RNA that is regulated by ER stress and protects oral cancer cells from ER stress‐induced apoptosis via promoting DNA repair.

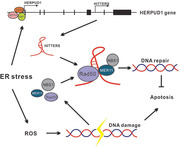

## 1 Introduction

The status of solid cancers, including oral squamous cell carcinoma (OSCC), inextricably links to stressful microenvironments such as hypoxia and lack of glucose or other nutrients.^[^ ^1^ ^]^ In response to endoplasmic reticulum (ER) stress, cells will activate unfolded protein response (UPR). Once the misfolded proteins are accumulated in ER lumen, the chaperone binding‐immunoglobulin protein (BIP) departs from the UPR sensors and activates three major UPR pathways that are IRE1*α*‐XBP1 pathway, Activating Transcription Factor (ATF) 6*α* pathway, and PRKR‐like ER kinase‐EIF2*α*‐ATF4 pathway. These UPR pathways govern various cellular events depending on the severity and duration of the stress. These cellular events include manipulation of gene transcription and translation, mRNA decay, inhibition of global protein synthesis, promotion of unfolded proteins degradation, and finally recovery of homeostasis or initiation of apoptosis.^[^ ^2^ ^]^

Evidence has indicated that DNA damage response (DDR) is crucial in OSCC carcinogenesis and treatment. Specifically, genes involved in DDR, such as *TP53*, *Ataxia Telangiectasia Mutated (ATM)*, and *Ataxia Telangiectasia And Rad3‐Related Protein (ATR)*, are frequently mutated in both OSCC tissues and patient‐derived OSCC tumoroids.^[^ ^3^ ^]^ Adjuvant chemoradiotherapy which triggers DDR is a routine treatment for OSCC patients.^[^ ^4^ ^]^ It is well established that ER stress is closely related to cancer cell proliferation, apoptosis, angiogenesis, and metastasis.^[^ ^1^ ^]^ However, little is known about the role of ER stress in DDR.

Long noncoding RNAs (lncRNAs) are a class of RNAs whose sequences are longer than 200 nucleotides and lack protein‐coding potential.^[^ ^5^ ^]^ LncRNAs regulate numerous cell activities in cancer via chromatin modification, transcriptional regulation, and post‐transcriptional regulation.^[^ ^6^ ^]^ Recent studies have proved that lncRNAs participated positively in DDR by sensing DNA damage, transducing damage signals, repairing damaged DNA, activating cell cycle checkpoints, and inducing apoptosis.^[^ ^7^ ^]^ Besides, researchers have discovered several lncRNAs that could regulate ER stress and UPR.^[^ ^8^, ^9^, ^10^ ^]^ However, little is known about how ER stress and UPR affect lncRNA transcription.

Given these issues, here we for the first time report the ER stress‐induced lncRNA transcriptomic changes in human OSCC cells and identifies thousands of differentially expressed lncRNAs. It is intriguing that only ≈10% of lncRNAs are selectively upregulated under ER stress, and among these upregulated genes the *HITTERS* (***H*** *ERPUD1* **i**n**t**ronic **t**ranscript of **ER s**tress), a novel lncRNA which is located in the sixth intron of *HERPUD1*, is the most altered lncRNA.

We next show that *HITTERS* is a nucleus‐located lncRNA including two transcript variants (TVs) which lack coding ability and are extremely sensitive to ER stress. As an intronic lncRNA, *HITTERS* lacks independent promoter and shares the same promoter with *HERPUD1*. Similar to *HERPUD1*, HITTERS is transcriptionally regulated by ATF6, XBP1s, ATF4, and DNA methylation. In human OSCC tissues, *HITTERS* shows strong coexpression with *HERPUD1* and is significantly correlated with OSCC clinicopathological features and prognosis.

Finally, functional experiments show that *HITTERS* acts as an oncogene to promote OSCC proliferation and invasion via influencing the expression of growth factors receptors and the downstream pathways. Under basal condition, *HITTERS* has no impact on apoptosis; however, once ER stress is triggered, *HITTERS* significantly attenuates ER stress‐induced apoptosis. Mechanically, we find that the ER stress‐induced antiapoptosis function of *HITTERS* dose not dependent on UPR initiation or *HERPUD1* expression. Instead, it functions as RNA scaffolds to promote MRE11‐RAD50‐NBS1 (MRN) complex formation to repair ER stress‐induced DNA damage.

## 2 Results

### 2.1 Identification of ER Stress‐Related lncRNAs

**Figure 1:**
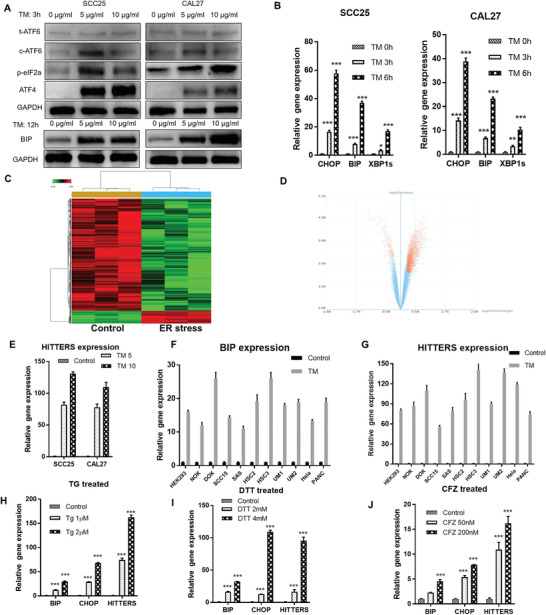
Identification of ER stress‐related lncRNAs. A) Western blot and B) qPCR confirmed that different concentrations of TM (5, 10 µg mL^−1^) or short time TM exposure could significantly induce ER stress of both SCC25 and CAL27 cells. C) Heatmap and D) volcano plot of HTA 2.0 lncRNA microarray for gene expression profiles of nontreated and TM‐treated SCC25 cells. E) qPCR confirmed the results of microarray that *HITTERS* was upregulated by treating cells with TM (5, 10 µg mL^−1^) for 6 h. F–J) qPCR showed F,G) treating different cells with TM (10 µg mL^−1^) for 6 h or H–J) treating SCC25 with different types of ER stress inducer for 6 h could upregulate both ER stress marker and *HITTERS*. The Student *t*‐test was used for analyzing the difference in (F) and (G). One‐way Analysis of Variance (ANOVA) test and Dunnett *t*‐test was used for (B), (E), and (H)–(J). For (E)–(G), all *p* < 0.001. Note: *, *P* < 0.05; **, *P* < 0.01, ***, *P* < 0.001.

So far, there is no lncRNA transcriptome analysis on the impact of ER stress in human cells. To explore this question, we treated human OSCC cell lines SCC25 and CAL27 with different concentration of tunicamycin (TM)^[^ ^11^ ^]^ and found that TM could significantly induce ER stress as determined by Western **Figure** **1**A). We then applied quantitative polymerase chain reaction (qPCR) to test the time‐course of TM induced ER stress markers and found that at the early phase (≤6 h), TM could significantly induce the expression of ER stress markers (Figure 1B).

Based on the above results, we used human transcriptome array 2.0 (HTA 2.0) microarray and analyzed the mRNA and lncRNA transcriptome change of TM treated SCC25 cells (5 µg mL^−1^, 6 h). 1070 (695 up, 375 down) differentially expressed mRNAs were found after TM treatment (*p *< 0.05, fold change ≥1.2). Both growth factor binding (GO) analysis and pathway analysis of differentially expressed mRNAs confirmed ER stress was significantly induced (Figure S1A,B, Supporting Information)

LncRNA expression profile indicated 2622 differentially expressed lncRNAs between TM treated and untreated cells, which consisted of 263 increased lncRNAs and 2359 decreased lncRNAs (*p *< 0.05, fold change ≥1.2; Figure 1C,D). These results indicated that at the early stage of ER stress, cells tended to repress the global lncRNA transcription, only a few lncRNAs were selectively upregulated. The transcript ENST00000570210.1 was the most significantly upregulated lncRNA under TM induced ER stress, which was further confirmed by qPCR (Figure 1E). ENST00000570210.1 is located on human chromosome 16: 56941028–56941726 forward strand, corresponding to the sixth intron of *HERPUD1*.

It is well known that lncRNA could regulate gene expression in cis and *HERPUD1* was ranked as the second most significant upregulated gene in the mRNA expression profile (Figure S1C, Supporting Information). Moreover, lncRNA‐mRNA coexpression network analysis showed that ENST00000570210.1 was highly correlated with multiple ER stress‐related genes (Figure S1D,E, Supporting Information). Therefore, we focused on ENST00000570210.1 and named it *HITTERS*.

We further treated another 11 human cell lines with TM. We also treated SCC25 and CAL27 with another three types of ER stress inducer, including thapsigargin (disturbing calcium flow of ER), dithiothreitol (DTT, disturbing disulfide bonds of ER), and carfilzomib (CFZ, disturbing proteasome activity). The results revealed that once ER stress was induced, *HITTERS* was significantly upregulated (Figure 1F–J), indicating that the upregulation of *HITTERS* under ER stress was a general phenomenon.

### 2.2 Basic Characteristics of *HITTERS*

**Figure 2:**
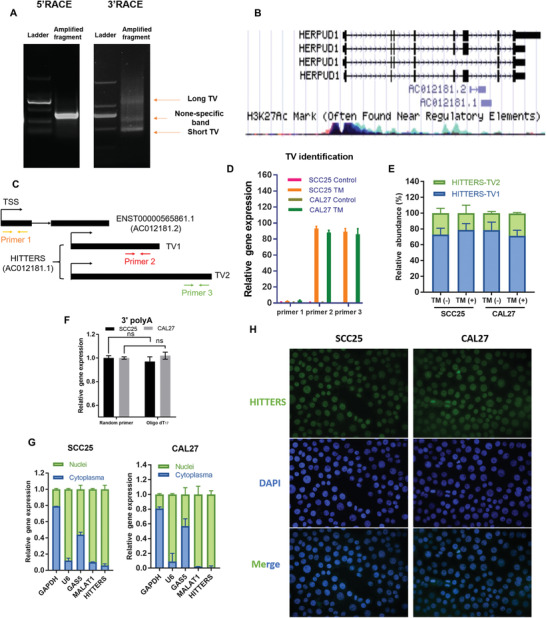
Basic characteristics of *HITTERS*. A) Agarose gel electrophoresis of PCR products generated by 5’ and 3’ RACE technologies. B) The schematic representation of *HERPUD1* and *HITTERS* (annotated as AC012181.1) in UCSC Genome Browser tracks. C) The schematic representation of qPCR primers design strategy for distinguishing two *HITTERS* TVs and *ENST00000565861.1*. D) qPCR showed two *HITTERS* TVs changed significantly in response to TM (10 µg mL^−1^, 6 h), whereas *ENST00000565861.1* did not change. Student *t*‐test was used. Only the changes of two *HITTERS* TVs were significant after TM treatment (*p* < 0.001). E) The relative abundance of two *HITTERS* TVs treated with or without TM (10 µg mL^−1^, 6 h). F) qPCR results on cDNA reverse‐transcripted by random primer or Oligo dT_17_ indicating that *HITTERS* had polyA tail. Student *t*‐test was used. “ns” stands for no significance. G) Nucleus‐cytoplasm fractionation qPCR and H) RNA FISH confirmed that *HITTERS* mainly located in cell nucleus.

Since that *HITTERS* is a new lncRNA that has not been fully characterized, we first analyzed its sequence using rapid amplification of cDNA ends (RACE) assay. The 5’‐RACE showed a clear single band, the 3’‐RACE showed 3 bands, one of which was an unspecific band (**Figure** **2**A). The RACE results revealed that *HITTERS* has 2 TVs. Compared to the 699 nt no‐variants lncRNA annotated in University of California Santa Cruz (UCSC) genome browser (Figure 2B), the *HITTERS* short TV (*HITTERS‐*TV1) is 793 nt, the *HITTERS* long TV (*HITTERS‐*TV2) is 1199 nt. Sequencing results mapped to the sixth intron of *HERPUD1*, namely the forward strand of chromosome 16: 56940933–56941725 (*HITTERS‐*TV1) and 56940933–56942131 (*HITTERS‐*TV2) (Table S4, Supporting Information). Two TVs shared the same 793 nt region and TV2 has an extra 406 nt 3’ arm (Figure 2C).

Another lncRNA *ENST00000565861.1* had an overlap region with *HITTERS* (Figure 2B). To differentiate these two genes, we designed specific primers (Figure 2C). qPCR results revealed that the expression of *ENST00000565861.1* remained unchanged after inducing ER stress (Figure 2D), which confirmed that *HITTERS* is an independent lncRNA.

We found two TVs of *HITTERS* were both highly responsive to ER stress (Figure 2C,D). The ratio of their expression levels remains no change, no matter the cells were under ER stress or not, and *HITTERS‐*TV1 was the predominant TV, which accounts for 75% of whole *HITTERS* TVs (Figure 2E). We also used random primer and oligo^[^ ^12^ ^]^_17_ primer to reverse transcribe the RNA and found the two primers showed equal efficiency (Figure 2F), indicating that *HITTERS* has a polyA tail.

Next, we cloned the sequence of *HITTERS* into pcDNA3.1(+)‐3xHA vector to explore its coding potential. None of the *HITTERS* recombinant plasmids showed a fusion protein (Figure S1F,G, Supporting Information), indicating that *HITTERS* lacks coding capability, no matter ER stress was triggered or not. Bioinformatic tools also predicted that similar to *HOX Transcript Antisense RNA (HOTAIR), HITTERS* had no protein coding ability (Figure S1H,I, Supporting Information). The subcellular location of lncRNA is very important for its function. By RNA fluorescent in situ hybridization (FISH) and nucleus‐cytoplasm fractionation qPCR, we found that *HITTERS* is predominantly located in the nucleus (Figure 2G,H). Overall, our results showed that *HITTERS* is a nuclear polyadenylated lncRNA with two TVs and *HITTERS‐* TV1 is the major TV.

### 2.3 *HITTERS* Shares the Same Promoter with *HERPUD1*

**Figure 3:**
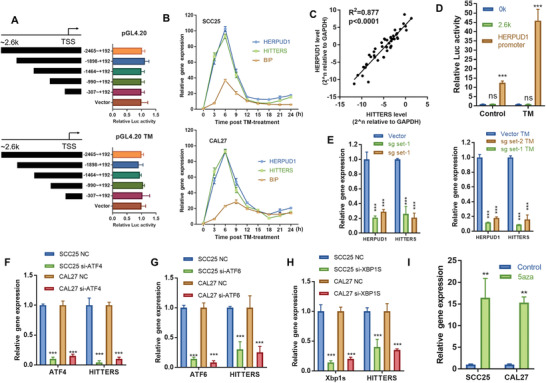
*HITTERS* shares the same promoter with *HERPUD1*. A) Dual‐luciferase reporter assay showed the potential promoter DNA fragments of *HITTERS* lacked transcription activity no matter threated with TM (10 µg mL^−1^, 6 h) or not. One‐way ANOVA and Dunnett *t*‐test were used, “Vector” was the control, all differences were none significant. B) qPCR results showed that time‐course change of *HITTERS* was identical to HERPUD1, but significant differed from BIP. Cells were treated with TM (10 µg mL^−1^) and measured every 3 h. C) qPCR results showed *HITTERS* and *HERPUD1* had a strong coexpression pattern in 48 OSCC samples. Liner‐regression test was used. D) Dual‐luciferase reporter assay showed the promoter of *HERPUD1* had strong transcription activity and responded obviously to TM (10 µg mL^−1^, 6 h). Student *t*‐test was used. E) qPCR results indicated that depleting *HERPUD1* promoter in HEK293 by CRISPR/Cas9 system significantly suppressed the expression level of both *HERPUD1* and *HITTERS*, no matter treated with or without TM (10 µg mL^−1^, 6 h). One‐way ANOVA and Dunnett *t*‐test were used, “Vector” was the control. qPCR results indicated knockdown of F) ATF4, G) ATF6, and H) XBP1s significantly suppressed *HITTERS* expression. Cells were transfected with siRNA for 48 h and then treated with TM (10 µg mL^−1^) for 6 h. Student *t*‐test was used. I) qPCR results showed treating cells with 5aza (10 × 10^−6^ m, 24 h) for inhibiting DNMTs significantly promoted *HITTERS* expression. Student *t*‐test was used. Note: ns, no significance; **, *P* < 0.01, ***, *P* < 0.001.

Since we have identified the chromosome location of *HITTERS*, we next explored how *HITTERS* was transcribed. We cloned the potential promoter region (−2465 to +192 of transcription start sites, TSS, ≈2.6 kb DNA fragment) of *HITTERS* into pGL4.20 vector and found none of these DNA fragments showed promoter activity, no matter whether the ER stress was induced or not (**Figure** **3**A). Another reporter vector pEZX‐FR01 showed identical results (Figure S1J,K, Supporting Information), which confirmed that *HITTERS* lacks independent promoter.

As *HITTERS* is an intron transcript of *HERPUD1*, we hypothesized that it shares the same promoter with *HERPUD1*. To test this hypothesis, we collected cells treated with TM every 3 h. qPCR results showed that the time‐course of changes in *HITTERS* and *HERPUD1* RNA levels were almost identical, whereas significantly differed from another ER stress marker *BIP* (Figure 3B). We also found that the expression of *HITTERS* and *HERPUD1* had a strong correlation in OSCC tissue samples (Figure 3C).

We then cloned the 338 bp promoter region (−303 to +35 of TSS) of *HERPUD1* as previously described^[^ ^13^ ^]^ and found this region had strong promoter activity and was highly responsive to ER stress (Figure 3D). We then applied the clustered regularly interspaced short palindromic repeats (CRISPR)/Cas9 technique and found after deleting this promoter region, the RNA levels of *HERPUD1* and *HITTERS* were both dramatically decreased (Figure 3E). Previous study has confirmed that this promoter region had ER stress response element (ERSE)‐I, ERSE‐II, and CCAAT/enhancer binding protein‐ATF composite cis‐acting element, to which ATF6, XBP1, and ATF4 bind and upregulate the transcription of *HERPUD1*.^[^ ^14^ ^]^ Also, DNA methylation was reported to suppress *HERPUD1* transcription.^[^ ^13^ ^]^ We found that similar to the transcription regulation of *HERPUD1*, silencing those transcript factors could decrease *HITTERS* RNA level (Figure 3F–H), whereas treating cells with DNA methyltransferase inhibitor 5aza could upregulate *HITTERS* RNA level (Figure 3I). Taken together, our results confirmed that *HITTERS* shares the same promoter with *HERPUD1*.

### 2.4 *HITTERS* Promotes OSCC Progression and Correlates with OSCC Clinicopathological Features

To explore the tumorigenic role of *HITTERS* in OSCC cells, we overexpressed *HITTERS‐*TV1 as it is the predominant TV. Since *HITTERS* mainly located in nucleus, we tested the knockdown efficiency of antisense oligonucleotide and siRNA and found siRNA is more effective. We selected one siRNA and constructed the corresponding shRNA lentivirus (Figure S2A–C, Supporting Information).

**Figure 4:**
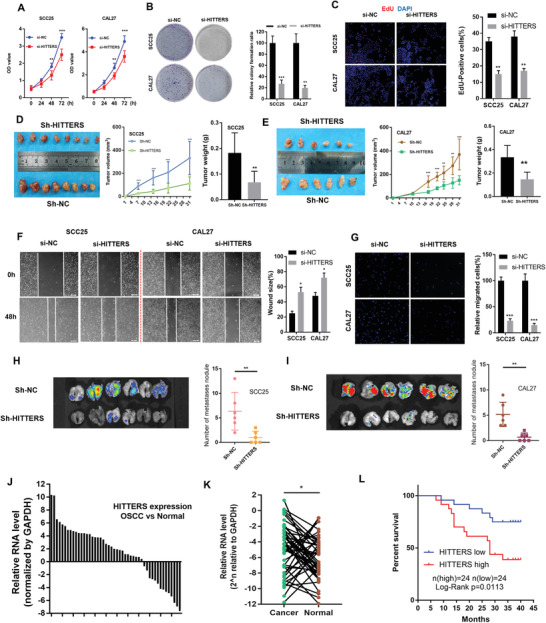
*HITTERS* promotes OSCC progression and correlates with OSCC clinicopathological features. A) CCK8, B) colony formation test, and C) EdU incorporation test confirmed that knockdown of *HITTERS* suppressed OSCC proliferation in vitro. D,E) Stably knockdown of *HITTERS* significantly suppressed tumor volume and tumor weight in D) SCC25 and E) CAL27 subcutaneous xenograft model. F) Wound healing test and G) transwell assay confirmed knockdown of *HITTERS* suppressed OSCC migration and invasion ability in vitro. Stably knockdown of *HITTERS* significantly suppressed H) SCC25 and I) CAL27 pulmonary metastasis nodule formation ability in vivo. The green fluorescent protein (GFP) fluorescense imaging of lungs was also presented. J) qPCR analyzed the relative fold of *HITTERS* in 48 OSCC tissues normalized by their paired adjacent normal tissues. K) qPCR results indicated the expression of *HITTERS* in OSCC was higher than their paired adjacent normal tissues. Paired *t*‐test was used. L) Kaplan–Meier curves showed high *HITTERS* expression had poor over‐all survival. Log‐rank test was used. For (A)–(I) Student *t*‐test was used. For (A), (D), and (E), Student *t*‐test was used for each time point. Note: *, *P* < 0.05; **, *P* < 0.01, ***, *P* < 0.001.

We found that knockdown of *HITTERS* significantly decreased cell proliferation, DNA replication, and colony formation in vitro (**Figure** **4**A–C). In contrast, overexpression of *HITTERS* dramatically increased cell proliferation, DNA replication, and colony formation (Figure S2D–F, Supporting Information). The stably transfected OSCC cells were subcutaneously inoculated into nude mice to investigate the function of *HITTERS* in vivo. Results showed tumors grown from *HITTERS* overexpression cells were significantly larger (Figure S2G,H, Supporting Information), whereas tumors from *HITTERS* stable knockdown cells were significantly smaller (Figure 4D,E), comparing to their own control cells, respectively.

The effects of *HITTERS* on the invasion ability of OSCC cells were evaluated using wound‐healing assay and transwell assay. The results showed that overexpression of *HITTERS* significantly promoted cell invasion (Figure 4F,G), while *HITTERS* knockdown inhibited invasion (Figure S2I,J, Supporting Information). We further examined the effects of *HITTERS* in lung colonization by injecting OSCC cells into the tail vein of mice. *HITTERS* overexpression significantly increased the development of lung metastases (Figure S2K,L, Supporting Information). Mice injected with *HITTERS* knockdown cells exhibited a marked reduction in lung metastases (Figure 4H,I).

Next, we analyzed the correlation between *HITTER* expression and OSCC patient's clinicopathological features. *HITTERS* was significantly upregulated in OSCC tissues compared to the corresponding adjacent normal tissues (Figure 4J,K). Moreover, high expression of *HITTERS* in OSCC is correlated with advanced T stage, positive lymph node metastasis, high‐risk clinical stage, and poor overall survival (Table S5, Supporting Information, and Figure 4L). These results suggested that *HITTERS* plays an important role in OSCC progression.

### 2.5 Receptor Tyrosine Kinases and Transforming Growth Factor (TGF)‐*β* Receptors Are Downstream Targets of *HITTERS*

To further uncover the mechanism of how *HITTERS* promoted OSCC progression, we performed RNA sequencing and gene set enrichment analysis (GSEA). *HITTERS* knockdown significantly influenced the GO, no matter ER stress was triggered or not (Figure S3A,B, Supporting Information), which indicating it is an intrinsic function of *HITTERS*. We used qPCR and confirmed that some important growth factor receptors, especially the receptor tyrosine kinases (RTKs) and TGF‐*β* receptors, were downregulated after knocking down *HITTERS* (Figure S3C,D, Supporting Information). Previous study has demonstrated that RTKs could promote cell proliferation via phosphatidylinositol 3‐kinase/protein kinase B (PI3K/AKT) and mitogen activated protein kinase (MAPK) pathway,^[^ ^15^ ^]^ and TGF‐*β* signaling pathway could promote cancer invasion via inducing Smad3 phosphorylation and subsequently epithelial‐mesenchymal transition (EMT).^[^ ^16^ ^]^ In accordance with these studies, we found that *HITTERS* knockdown significantly decreased phosphorylation of AKT, ERK1/2, and Smad3 (Figure S3E, Supporting Information). Moreover, many downstream genes involved in cell proliferation and EMT were also regulated by *HITTERS* (Figure S3F,G, Supporting Information). Taken together, our results suggested that *HITTERS* promotes OSCC progression potentially via regulating RTKs and TGF‐*β* pathway.

### 2.6 *HITTERS* Attenuates ER Stress Induced Apoptosis

**Figure 5:**
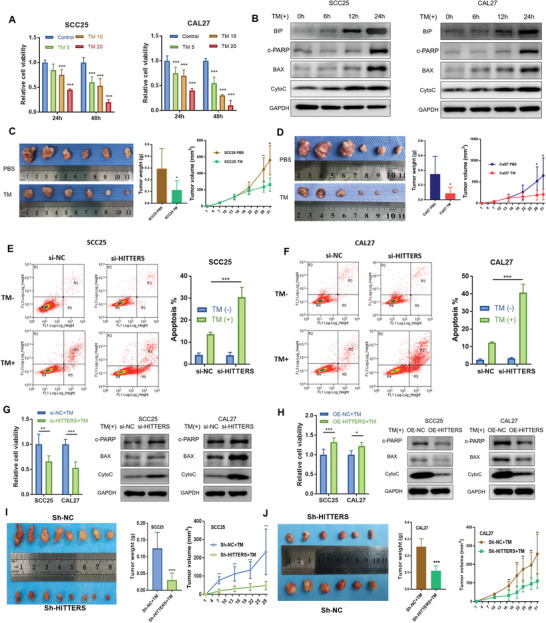
*HITTERS* attenuates ER stress induced apoptosis. A) Cell viability (measured by CCK8) of SCC25 and CAL27 were significantly suppressed by TM (5, 10, and 20 µg mL^−1^ for 24 and 48 h) in a dose‐ and time‐dependent manner. One‐way ANOVA and Dunnett *t*‐test were used for each time point. B) Western blot showed the ER stress marker BIP and apoptosis marker of SCC25 and CAL27 cells were significantly upregulated by TM (10 µg mL^−1^) in a time‐dependent manner. c‐PARP, cleaved PARP. C,D) Intraperitoneally injection of TM twice a week significantly suppressed tumor volume and tumor weight in C) SCC25 and D) CAL27 subcutaneous xenograft model. E,F) Under non‐ER stress condition, *HITTERS* would not affect apoptosis; however, depletion of *HITTERS* in TM (10 µg mL^−1^, 24 h) treated E) SCC25 and F) CAL27 cells significantly promoted apoptosis. Apoptosis was measured by Annexin‐V/PI double staining and flow cytometry. Proportion of R3 + R5 is considered apoptosis. Cells were transfected with siRNA for 48 h and then treated with TM (10 µg mL^−1^, 24 h). Two‐way ANOVA and Sidak's multiple comparisons test were used. G) Knocking‐down *HITTERS* significantly suppressed cell viability and promoted apoptosis marker expression; H) whereas overexpressing *HITTERS* obtained the opposite effect. Cells were transfected with siRNA for 48 h and then treated with TM (10 µg mL^−1^, 24 h). Cell viability differences were measured by CCK8 using Student *t*‐test. Stably knockdown of *HITTERS* causing I) SCC25 and J) CAL27 more sensitive to ER stress in vivo, reflected by a significantly reduction in tumor volume and tumor weight in subcutaneous xenograft model. All BALB/c nude mice were intraperitoneally injected with TM, twice a week, after tumor bearing. Student *t*‐test was used. Note: *, *P* < 0.05; **, *P* < 0.01, ***, *P* < 0.001; ****, *P* < 0.0001.

Severe or prolonged ER stress finally ends up with apoptosis. Since *HITTERS* is dramatically increased under ER stress, we explored its role in ER stress‐induced apoptosis. We found that TM treatment significantly decreased cell viability in a time‐ and dose‐dependent manner in OSCC cells (**Figure** **5**A). The expression of apoptotic markers such as cleaved poly(ADP‐Ribose) polymerase (c‐PARP), BCL2 associated X, and cytochrome C were significantly increased after exposure to TM (Figure 5B). In order to test the effects of TM in vivo, mice were intraperitoneally injected with TM. We found that TM could significantly inhibited OSCC growth in vivo (Figure 5C,D). These results suggest that ER stress could induce OSCC apoptosis.

We then tested whether *HITTERS* regulates ER stress induced apoptosis. Via Annexin V‐fluorescein isothiocyanate/propidium iodide (FITC/PI) staining, we found that *HITTERS* knockdown did not influence apoptosis under basal condition. Once ER stress was triggered, depletion of *HITTERS* significantly promoted apoptosis and decreased cell viability. In contrast, overexpression of *HITTERS* increased cell viability under ER stress (Figure 5E–H). We also found that *HITTERS* knockdown promoted the expression of apoptotic markers, whereas overexpression of *HITTERS* inhibited their expression (Figure 5G,H). In agreement with the in vitro studies, tumors grown from *HITTERS* overexpression cells were more resistant to ER stress induced apoptosis (Figure S4A,B, Supporting Information), whereas tumors from *HITTERS* knockdown cells were sensitive to ER stress induced apoptosis (Figure 5I,J). Taken together, these results showed that *HITTERS* could attenuate ER stress induced apoptosis.

### 2.7 *HITTERS* Regulates ER Stress Related DNA Damage Response

**Figure 6:**
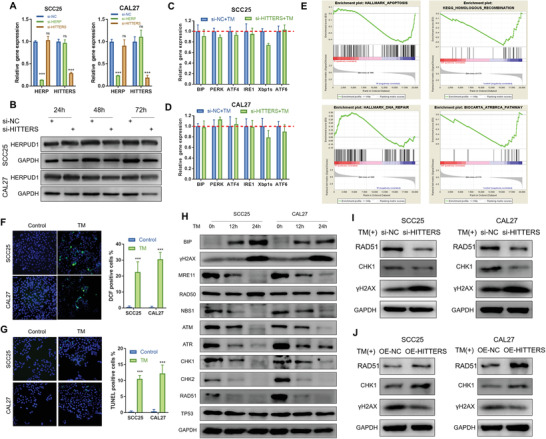
*HITTERS* regulates ER stress related DDR. A) qPCR and B) Western blot confirmed that knocking‐down *HITTERS* did not significantly change the mRNA and protein expression of *HERPUD1*. For qPCR, cells were transfected with siRNA for 48 h. Student *t*‐test was used for qPCR. C,D) Knocking‐down *HITTERS* did not significantly change the mRNA level of important UPR regulator in both C) SCC25 and D) CAL27 cells. Cells were transfected with siRNA for 48 h and then treated with TM (10 µg mL^−1^) for 6 h. E) GSEA results for RNA‐sequencing profiles. SCC25 cells were transfected with *HITTERS* siRNA or negative control siRNA for 48 h and then treated with TM (10 µg mL^−1^) for 12 h. F,G) 2',7'‐dichlorofluorescein staining and TUNEL assay indicated TM (10 µg mL^−1^, 24 h) treatment promoted F) ROS production and G) DNA breaks. Student *t*‐test was used. H) Induction of ER stress by TM (10 µg mL^−1^) significantly promoted the level of DNA damage marker *γ*‐H2AX and suppressed DNA repair proteins in a time‐dependent manner. I) Knocking‐down *HITTERS* significantly suppressed DNA repair protein and promoted DNA damage marker expression; J) whereas overexpressing *HITTERS* obtained the opposite effect. Cells were transfected with siRNA or plasmid for 48 h and then treated with TM (10 µg mL^−1^) for 24 h. Note: ns, no significance; ***, *P* < 0.001.

Next, we investigated how *HITTERS* regulates ER stress induced apoptosis. We found that *HITTERS* knockdown did not influence the mRNA and protein levels of *HERPUD1* (**Figure** **6**A,B). Moreover, *HITTERS* knockdown had little influence on the expression of important UPR molecules (Figure 6C,D). These results indicated that *HITTERS* may not regulate ER stress induced apoptosis through regulating *HERPUD1* in cis or UPR initiation in trans. We then performed RNA sequencing and GSEA analysis. GSEA showed that *HITTERS* had a significant impact on OSCC apoptosis under ER stress (Figure 6E). More importantly, we found that under ER stress, depletion of *HITTERS* significantly affected DDR, especially the DNA repair related pathways, including homologous recombination (HR) pathway and ATR‐breast cancer susceptibility genes (BRCA) pathway (Figure 6E). Unrepaired DNA damage, especially double strand breaks, is one of the most cytotoxic DNA lesions which leads to cell death through apoptosis.^[^ ^17^ ^]^ Since we have found *HITTERS* significantly inhibited apoptosis, we next focused on exploring the functional role of *HITTERS* in ER stress‐related DDR.

Previous study has shown that ER stress could induce reactive oxygen species (ROS) production and cause oxidative DNA damage.^[^ ^18^ ^]^ We found that ER stress could significantly trigger ROS production (Figure 6F). Terminal deoxynucleotidyl transferase dUTP nick end labeling (TUNEL) assay directly proved that ER stress could induce DNA strand breaks (Figure 6G). Consequently, we found ER stress induced a significant upregulation of DNA damage marker *γ*‐H2AX (Figure 6H). HR and nonhomologous end joining (NHEJ) are two major pathways for repairing double strand breaks.^[^ ^19^ ^]^ We then examined the expression of HR and NHEJ pathways under ER stress. We found that most HR related proteins, including MRE11, NBS1, ATM, ATR, CHK1, CHK2, and RAD51, were dramatically downregulated in a time‐dependent manner, except for RAD50 and TP53, whose expression was unaltered (Figure 6H). In contrast, we found proteins related to NHEJ remained unchanged under ER stress (Figure S4C, Supporting Information).

Next, we investigated how *HITTERS* affected ER stress related DDR. We found that changing the expression level of *HITTERS* did not influence ROS production (Figure S4D, Supporting Information). However, depletion of *HITTERS* significantly increased the ratio of TUNEL positive cells (Figure S4E, Supporting Information) and the level of *γ‐H2AX* (Figure 6I), whereas overexpression of *HITTERS* suppressed TUNEL positive ratio (Figure S4E, Supporting Information) and *γ*‐H2AX expression (Figure 6J). Moreover, *HITTERS* was positively associated with the expression of RAD51 and CHK1 that are involved in HR (Figure 6I,J). However, *HITTERS* had no influence on NHEJ pathway (Figure S4C, Supporting Information). Taken together, our findings suggested that ER stress could simultaneously induce oxidative DNA damage and suppress HR via downregulation of the crucial proteins. *HITTERS* acted as a protective factor in ER stress induced DNA damage, but the protective effect was not through regulating ROS production.

### 2.8 *HITTERS* Binds to and Promotes the Formation of MRE11/RAD50/NBS1 Complex under ER Stress

**Figure 7:**
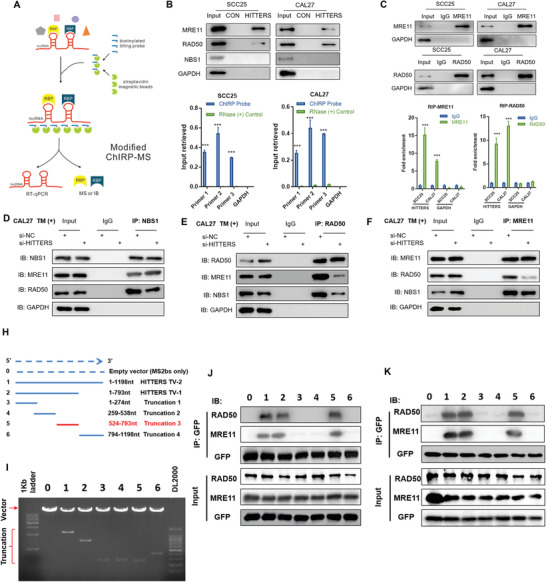
*HITTERS* binds to and regulates the formation of MRN complex under ER stress. A) The schematic representation of modified ChIRP‐MS. B) qPCR showed the modified ChIRP method retrieved about 30–50% of *HITTERS*. Western blot confirmed that *HITTERS* binds to both MRE11 and RAD50, but not NBS1 nor glyceraldehyde‐3‐phosphate dehydrogenase. Cells were treated with TM (10 µg mL^−1^, 6 h) before harvesting for ChIRP‐MS. For qPCR, Student *t*‐test was used for each primer. ***, *P* < 0.001. C) RIP assay of MRE11 and RAD50. Western blot confirmed MRE11 and RAD50 were successfully precipitated. qPCR indicated *HITTERS* were significantly enriched by MRE11 and RAD50. Cells were treated with TM (10 µg mL^−1^, 6 h) before harvesting for RIP. For qPCR, Student *t*‐test was used. ***, *P* < 0.001. D,E) Co‐IP results showed the interaction between MRE11 and RAD50 in CAL27 cells were reduced after *HITTERS* knockdown. The interaction between MRE11 and NBS1 remained no change after *HITTERS* knockdown. Cells were transfected with siRNA for 48 h and then treated with TM (10 µg mL^−1^) for 6 h. H) Diagrams of full‐length *HITTERS* and the truncations in MS2bs‐MS2bp RNA pull‐down assay. I) The reconstructed plasmids containing 12XMS2 tag and full‐length HITTERS and truncations with the correct sizes are indicated. J,K) Immunoblot analysis for RAD50 and MRE11 in the protein samples pulled down by different HITTERS truncations. J) SCC25 and K) CAL27 cells were transfected with two plasmids for 48 h and treated with TM (10 µg mL^−1^) for 6 h.

To identify the endogenous proteins that interact with *HITTERS* under ER stress, a modified comprehensive identification of RNA‐binding proteins by mass spectrometry (ChIRP‐MS) method was used (**Figure** **7**A). The antisense probe pool retrieved about 30–50% of total *HITTERS* (Figure 7B). Liquid chromatograph‐MS/MS result indicated that 47 proteins, including RAD50 and MRE11, interacted with *HITTERS* (File S1, Supporting Information). Western blotting confirmed that under ER stress, *HITTERS* interacted with both RAD50 and MRE11 (Figure 7B). Interestingly, NBS1, a member of MRN complex, was not enriched by *HITTERS* (Figure 7B). We next performed RNA immunoprecipitation (RIP) assay and found both RAD50 and MRE11 bind to *HITTERS* (Figure 7C). These results confirmed that *HITTERS* endogenously interacted with RAD50 and MRE11.

MRN has a central function in DNA damage repair by sensing the damaged DNA, processing broken DNA ends and activating DNA damage repair pathways.^[^ ^17^, ^20^, ^21^ ^]^ We found depletion of *HITTERS* significantly decreased the protein level of MRE11 and NBS1 but had no effect on RAD50 expression (Figure S5A, Supporting Information). In contrast, the application of Mirin, a small molecule inhibitor of MRE11, or *RAD50*‐specific siRNA, could not change *HITTERS* expression (Figure S5B, Supporting Information). Therefore, *HITTERS* may act as an upstream regulator of the MRN complex. We then explored how *HITTERS* influence MRE11 and NBS1 protein level. We found that *HITTERS* had no impact on the transcription of MRE11 and NBS1, as the RNA level of MRE11 and NBS1 did not significantly change with *HITTERS* gain or loss of function (Figure S5C, Supporting Information). ER‐stress could suppress global protein translation via eif2*α* phosphorylation. However, we found that knockdown of *HITTERS* had no impact on eif2*α* phosphorylation and directly mimicking eif2*α* phosphorylation via Salubrinal treatment did not influence the protein level of NBS1 and MRE11 (Figure S5D, Supporting Information). Finally, we hypothesized that *HITTERS* might influence the protein degradation of NBS1 and MRE11. Our results proved that under ER‐stress, NBS1 and MRE11 were degraded via ubiquitin‐proteasome system rather than autophagy system (Figure S5E,F, Supporting Information). Depletion of *HITTERS* significantly promoted the ubiquitination of MRE11 and NBS1, and inhibiting proteasome could rescue the degradation (Figure S5I,J, Supporting Information).

We also hypothesized that *HITTERS* could influence the interaction between RAD50 and MRE11. We treated cells with TM for 6 h as MRE11 and NBS1 were not significantly degraded at this time‐point (Figure S4C, Supporting Information). Co‐immunoprecipitation (co‐IP) analysis (Figure 7D–F and Figure S6A–C, Supporting Information) showed that RAD50, MRE11, and NBS1 were tightly interacted with each other under ER stress. However, after knocking‐down of *HITTERS*, the interaction between RAD50 and MRE11 was significantly weakened. However, depletion of *HITTERS* did not influence the interaction between MRE11 and NBS1. These results indicated that *HITTERS* promoted MRN complex formation via facilitating the interaction between MRE11 and RAD50.

The binding domain of MRE11 and RAD50 in this structure is well documented.^[^ ^21^ ^]^ Therefore, we speculate that *HITTERS* could interact with the domain. However, the binding sites of *HITTERS* with MRN need to be validated. To explore this, we used MS2bs‐MS2bp RNA pull down assay. We established a serial *HITTERS* RNA truncation and found that 524–793nt of *HITTERS* mediated its association with RAD50 and MRE11.

Since the function of MRN complex in ER stress‐related DDR is not well understood, we used siRNA or Mirin to inhibit MRN complex function. Inhibited of MRN complex resulted in elevated levels of DNA damage in OSCC, which was comparable to the levels found in *HITTERS* knockdown cells (Figure S4E, Supporting Information). Correspondingly, the expression of DNA damage marker *γ*‐H2AX and proapoptotic proteins were upregulated by *HITTERS* siRNA, *RAD50* siRNA or Mirin, whereas cell viability and the DNA damage repair proteins RAD51 and CHK1 were downregulated (Figure S6D,E, Supporting Information). Overexpression of *HITTERS* enhanced cell viability by inhibiting DNA damage and promoting DNA repair. However, the protective effects were markedly abolished by dysfunction of MRN complex induced by *RAD50* siRNA and Mirin (Figure S6F,H, Supporting Information). Taken together, our results showed that *HITTERS* functions as an RNA scaffold to promote MRE11‐RAD50 interaction and increase the protein levels of MRE11 and NBS1, also, the antiapoptosis function of *HITTERS* relies on MRN complex.

## 3 Discussion

ER stress‐triggered UPR is initially activated as a prosurvival mechanism by maintaining ER homeostasis and promoting protein folding and degradation in response to environmental challenges. However, severe or prolonged ER stress ultimately leads to cell death. Therefore, it is important to find out the gene that determines the final effects of ER stress. In the present study, we uncovered a novel lncRNA *HITTERS* by comprehensive characterization of the ER stress related lncRNA transcriptome. Only ≈10% of lncRNAs were selectively upregulated under ER stress and *HITTERS* was the most significantly upregulated lncRNA. We showed that *HITTERS* promoted proliferation and invasion of OSCC both in vitro and in vivo. *HITTERS* expression was upregulated in OSCC tissues and the upregulation of *HITTERS* was correlated with poor survival of patients with OSCC. More importantly, our findings demonstrated that *HITTERS* significantly inhibited ER stress induced apoptosis. Mechanistically, *HITTERS* could bind to and regulate the formation of MRN complex, and increase the expression of proteins involved in DNA damage repair, therefore alleviating ER stress‐related DNA damage. These findings underscore the potential of *HITTERS* as a novel target for cancer therapy.

Recent advances in RNA sequencing technology allow researchers to discover thousands of lncRNAs in eukaryotic genomes. However, the function and molecular mechanism of the majority of lncRNAs in human disease, especially in cancer, remain elusive. Many lncRNAs are reported to regulate proliferation, survival, angiogenesis, and invasion through modulating PI3K/Akt and ERK1/2 MAPK pathways.^[^ ^22^ ^]^ Our data showed that under non‐ER stress condition, *HITTERS* promoted the phosphorylation of Akt and ERK1/2, and the expression of downstream targets proliferating cell nuclear antigen, Cyclin D1, and P27 was also regulated. Moreover, we found that *HITTERS* activated TGF‐*β*/Smad3 pathway, promoted transcription factors Snail and Slug, leading to increased mesenchymal markers such as Vimentin and *N*‐cadherin and decreased epithelial marker E‐cadherin. TGF‐*β* is a well‐known inducer of EMT during cancer progression, and many lncRNAs could regulate its expression. For example, lncRNA *ANRIL* promotes the invasion and metastasis in OSCC, prostate cancer, and thyroid cancer through regulating TGF‐*β*/Smad3 pathway.^[^ ^23^ ^]^ LncRNA *ELIT‐1* behaves as a Smad3 cofactor to facilitate TGF‐*β*/Smad3 pathway and induce EMT.^[^ ^24^ ^]^ Therefore, the effects of *HITTERS* on proliferation and invasion are partly attributed to the activation of PI3K/Akt, ERK1/2 MAPK, and TGF‐*β*/Smad3 pathway.

As the functions of lncRNAs in physiological and pathological processes have become increasingly recognized, the association of lncRNAs with ER stress attracts more attention. To date, a few lncRNAs such as *HypERlnc*,^[^ ^10^ ^]^ *TUG1*,^[^ ^25^ ^]^ and *MEG3* ^[^ ^9^ ^]^ are reported to be associated with ER stress. In this study, we demonstrated that ER stress significantly increased *HITTERS* expression. Elevated level of *HITTERS*, in return, inhibited ER stress induced apoptosis. Therefore, cancer cells may utilize *HITTERS* to overcome ER stress induced apoptosis in adverse environment. We next analyzed the mechanism of *HITTERS* regulated ER stress. In striking contrast to *HypERlnc*, *TUG1*, and *MEG3*, neither *BIP* expression, nor the three main UPR pathway, were affected by *HITTERS*. These results suggest that *HITTERS* modulates ER stress induced apoptosis through a novel mechanism. ER stress was reported to inhibit DNA repair by proteasomal degradation of RAD51.^[^ ^26^ ^]^ Indeed, we found that ER stress promotes ROS production to cause DNA damage, and at the same time suppresses DNA repair, finally leading to apoptosis. Most importantly, we demonstrated that although *HITTERS* could not inhibit ROS production, it could significantly enhance DNA damage repair pathways under ER stress, thus protects OSCC from ER stress induced apoptosis.

Recent studies have proved that lncRNAs participated positively in DDR through varieties of mechanism.^[^ ^7^ ^]^ LncRNAs can act as RNA scaffolds to regulate DDR. For example, lncRNA *GUARDIN* sustains *BRCA1* stability by facilitating the *BRCA1‐BARD1* complex. In this study, our work for the first time found that inhibiting MRN complex accelerated ER stress induced DNA damage and apoptosis. We demonstrated that *HITTERS* facilitated the formation of MRN complex under ER stress. If *HITTERS* was knocked down, the binding capacity between *MRE11* and *RAD50* was disturbed. Inhibiting MRN complex function abolished the protective effect of *HITTERS*, indicating that the function of *HITTERS* relies on MRN complex. It has been acknowledged that MRN can assemble as a heteromultimer, and the DNA binding and processing structure of the heteromultimer is formed by the *MRE11* dimer and two *RAD50*.^[^ ^21^ ^]^ The binding domain of MRE11 and RAD50 in this structure is well documented. Therefore, we speculate that HITTERS could interact with the domain. Our results found 24‐793 nt of *HITTERS* mediated its association with RAD50 and MRE11. Our results also showed that depletion of *HITTERS* significantly decreased the protein level of MRE11 and NBS1, via promoting proteasomal degradation of MRE11 and NBS1, even though *HITTERS* did not interact with NBS1. Thus, we hypothesized that under ER stress, *HITTERS* might influence the proteasome activity. However, the mechanism needs further exploration.

Our results found that ER‐stress could significantly increase *HITTERS* expression, in contrast, the DNA damage repair proteins decreased dramatically. Further analysis found if the *HITTERS* was depleted, the DNA damage repair proteins would decrease more significantly. These data indicated that the increment of HITTERS by TM treatment could be a protective feedback. It is intriguingly that *HITTERS* shares the same promotor with *HERPUD1*. However, we found that *HITTERS* knockdown does not influence the mRNA and protein levels of *HERPUD1*. This raises doubt on why they share the same promoter? Whether *HERPUD1* could act like *HITTERS*, by regulating PI3K/Akt, ERK1/2 MAPK, and TGF‐*β*/Smad3 pathway, or participating ER stress induced DDR, are still unclear. It is reasonable that the function of these two genes may cooperate as their transcription regulation are identical. Another possible explanation is that *HITTERS* is independently processed from the pre‐mRNA of *HERPUD1*. In support of this hypothesis, our ChIRP‐MS results showed *HITTERS* could pull‐down DDX39B which is closely related to pre‐mRNA splicing. However, further studies will be necessary to verify this hypothesis.

To sum up, this study presents a novel lncRNA, namely *HITTERS*, which links ER stress and DDR together in OSCC. Mechanisms presented here could provide viable therapeutic choices in remedying stressful microenvironment‐associated tumor progression.

## 4 Experimental Section

##### Cell Culture, Transfection, and Chemical Treatment

Human OSCC cell lines SCC25 and CAL27 were purchased from American Type Culture Collection. Other cell lines including HEK293 (human embryonic kidney cell line), Hela (human cervical cancer cell line), PANC‐1 (human pancreatic cancer cell line), NOK (human normal oral keratinocytes), DOK (human dysplastic oral keratinocytes), and other human OSCC cell lines (SAS, UM‐1, UM‐2, SCC15, HSC‐2, and HSC‐3) were all obtained from State Key Laboratory of Oral Diseases (Sichuan University, China). SCC25, SAS, UM‐1, UM‐2, and SCC15 were cultured in Dulbecco's Modified Eagle Medium / Nutrient Mixture F‐121:1, other cell lines were cultured in Dulbecco's Modified Eagle Medium. All medium was supplemented with 10% fetal bovine serum (Gibco). All cell lines were cultured at 37 °C with 5% CO_2_. Cell lines were authenticated by short tandem repeat profiling. All cell lines were free for mycoplasma contamination. For transient transfection of siRNA (GenePharma) or plasmid, the EndoFectin (Genecopoeia) was applied according to the manufacture's instruction. For stable transfection, the lentivirus (Genecopoeia) containing shRNA or lncRNA sequence plasmid was applied according to the manufacture's instruction. For inducing ER stress, TM (5–20 µg mL^−1^, Sigma), thapsigargin (Tg, 1 × 10^−6^–2 × 10^−6^ m, MedChemExpress), DTT (2 × 10^−3^–4 × 10^−3^ m, Takara), and CFZ (50 × 10^−9^–200 × 10^−9^ m, Selleck) were added. For inhibiting DNA methyltransferase, 5aza (10 × 10^−6^ m, Sigma) were added for 24 h. For inhibiting MRN complex, Mirin (100 × 10^−6^ m, Selleck) were added simultaneously with other treatment. For inhibiting proteasome, MG‐132 (10 × 10^−6^ m, Beyotime) were added for 12 h. For inhibiting autophagy/lysosome, hydroxychloroquine sulfate (100 × 10^−6^ m, Selleck) was added for 48 h. For mimicking eif2a phosphorylation, Salubrinal (50 × 10^−6^ m, Selleck) was added for 24 h.

##### ChIRP‐MS

ChIRP‐MS was carried out following Chu's protocol^[^ ^27^ ^]^ with several modification. For each reaction, 5 × 10^7^ cells were harvested. Micrococcal nuclease (New England Biolabs (NEB)) was used for DNA fragmentation followed by ultrasonication. The 3′‐biotin‐triethylene glycol modified ChIRP probes (Sangon) were added to streptavidin‐coated magnetic beads (Biomeg), washed, and then hybridized with cell lysates. An excessive dose of probes was used (tenfold higher concentrations). For protein extraction, the trichloroacetic acid precipitation step was skipped.

##### Dual‐Luciferase Reporter Assay

The targeted inserting sequence was amplified by PCR using Q5 High‐Fidelity DNA Polymerase (NEB). Quickcut (Takara) restriction endonucleases and DNA Ligation Kit (Takara) was used for vector reconstruction. The full‐length (2.5 kb around TSS) and truncated sequence of potential promoter regions were amplified by PCR. PCR products were then inserted into luciferase reporter plasmids pGL4.20 (Luc2, Promega) or pEZX‐FR01 (containing both Fluc and Rluc, Genecopoeia). When using pGL4.20, the pGL4.75 plasmid (Rluc) was cotransfected as an internal control. The dual‐luciferase reporter assay was applied in 96‐well palates at 5 × 10^4^ cells per well. HEK293T cells were transfected with 500 ng of plasmid. Luciferase activity was determined using Luc‐Pair Duo‐Luciferase HS Assay kit (Genecopoeia).

##### Generation of CRISPR/Cas9 Construct

The sgRNA targeting the promoter region of *HERPUD1* was designed by the online design tool Crispor. After phosphorylating and annealing of each pair of oligos using T4 polynucleotide kinase (Thermo), the oligo was inserted into BsmBI (Thermo)‐digested LentiCRISPRV2 vector (Addgene). The forward and reverse oligo pair was inserted into vectors containing puromycin and neomycin resistance, respectively. The reconstructed vector was transfected into HEK293 and selected for 2 weeks to obtain *HERPUD1* promoter knockout cell lines.

##### High‐Throughput Analysis

Total RNA was extracted from SCC25 cells using Trizol reagent (Invitrogen). The RNA samples were subjected to mRNA and lncRNA microarray using GeneChip HTA 2.0 (Affymetrix) at Genminix Informatics Ltd., Co. HTA 2.0 covers more than 245 000 coding transcripts and 40 000 noncoding transcripts in human transcriptome. Gene Ontology (GO) analysis, pathway analysis, and lncRNA‐mRNA coexpression network analysis of the differentially expressed mRNAs and lncRNAs were performed as previously described.^[^ ^28^ ^]^ Next generation sequencing of mRNA transcriptome and GSEA were carried out as previously described (OE biotech, China) in SCC25 cells.^[^ ^29^ ^]^

##### Animal Study

All animal studies were approved by the Animal Ethical and Welfare Committee of West China Hospital of Stomatology (WCCSIRB‐D‐2016‐075). Female BALB/C nude mice were maintained under specific pathogen free conditions in the Sichuan University Animal Center according to the institution's guidelines. For subcutaneous xenograft model, 5 × 10^6^ OSCC cells in 200 µL phosphate buffered saline (PBS) were sub‐axillary injected. The tumor volume was documented in indicated time. For in vivo TM treatment, TM were diluted by PBS to 20 µg mL^−1^. Mice were intraperitoneally injected with 100 µL of PBS or TM, twice a week. At the 4th week postinjection, animals were sacrificed, and the tumors were harvested. For pulmonary metastasis model, 1 × 10^6^ OSCC cells in 100 µL PBS were injected into the tail vein. At the 5th week postinjection, the animals were sacrificed and the lungs were harvested.

##### Patients’ Samples

The Institutional Ethical Committee of West China Hospital of Stomatology approved the present study (WCHSIRB‐OT‐2016‐047). All patients have signed written informed consent. All procedures were carried out in accordance with the Declaration of Helsinki. Human oral cancer tissue specimens were collected from the West China Hospital of Stomatology, Sichuan University (China). A total of 48 primary OSCC samples and paired adjacent (>1.5 cm from the tumor margin) normal tissues were obtained. After resection, the samples were snap frozen by liquid nitrogen and stored in −80 °C for up to 1 week before qPCR analysis.

##### Additional Methods

Cell counting kit‐8 (CCK‐8), EdU incorporation, colony formation assay, apoptosis, wound healing assay, cell invasion, TUNEL, ROS detection, nucleus‐cytoplasm fractionation, RNA FISH, RACE, RNA extraction and qPCR, Western blotting, co‐IP, RIP, and MS2bs‐MS2bp RNA pull down are detailed in the Supporting Information. All the primers, probes, oligos, and antibodies are listed in Table S1–S3 in the Supporting Information.

##### Statistical Analysis

GraphPad Prisma 7 software was used for statistical analysis and generating statistical figures. Data were presented from one representative experiment out of three independent experiments. For each representative experiment, at least three repetitions were measured. The statistical methods for each result were noted in figure legend. Data were presented as mean ± standard deviation. *p* < 0.05 was considered statistically significant.

## Conflict of Interest

The authors declare no conflict of interest.

## Supporting information

Supporting Information

Supporting Information
