# Supplementary material for: Long Noncoding RNA HITTERS Protects Oral Squamous Cell Carcinoma Cells from Endoplasmic Reticulum Stress‐Induced Apoptosis via Promoting MRE11‐RAD50‐NBS1 Complex Formation
Source: Adv Sci (Weinh). 2020 Oct 4;7(22):2002747. doi: 10.1002/advs.202002747 (PMC7675039; doi:10.1002/advs.202002747)
Supplement: Supplementary file 1 — Supporting Information [file ADVS-7-2002747-s001.pdf]

© 2020 Wiley-VCH GmbH

ADVANCED HEALTHCARE MATERIALS

Supporting Information

for *Adv. Healthcare Mater.*, DOI: 10.1002/adhm.202002747

Long Noncoding RNA *HITTERS* Protects Oral Squamous Cell Carcinoma Cells from Endoplasmic Reticulum Stress-Induced Apoptosis via Promoting MRE11-RAD50-NBS1 Complex Formation

Chenzhou Wu, Wen Chen, Fanyuan Yu, Yihang Yuan, Yafei Chen, Douglas R. Hurst, Yi Li, Longjiang Li, Zhe Liu**

Supporting Information

Long Noncoding RNA *HITTERS* Protects Oral Squamous Cell Carcinoma Cells from Endoplasmic Reticulum Stress-Induced Apoptosis via Promoting MRE11-RAD50-NBS1 Complex Formation

Chenzhou Wu, Wen Chen, Fanyuan Yu, Yihang Yuan, Yafei Chen, Douglas R. Hurst, Yi Li, Longjiang Li*, Zhe Liu*

Supplementary Experimental Section

CCK-8 assay: Proliferation of OSCC cells were evaluated using a CCK-8 assay (Dojindo). SCC25 and CAL27 cells were plated in 96-well plates at a density of 5×10^3 cells per well. After culturing at 37 °C for 24, 48, 72, and 96 h, cells were treated with 10% CCK-8 for 1 h. The absorbance of each well was measured with a microplate reader at 450 nm.

EdU incorporation assay: SCC25 and CAL27 cells were seeded in 48-well plates at a density of 3×10^4 cells per well and were allowed to adhere overnight in complete medium. Cells were incubated with 5-ethynyl-2'-deoxyuridine (EdU, Genecopoeia) for 2 h, followed by fixation, permeabilization, and EdU staining. Nuclei were counterstained with Hoechst 33342 (5 μg/ml) for 15 min. Percentage of proliferative cells (EdU-positive) were counted under a fluorescence microscope (Olympus).

Colony formation assay: SCC25 and CAL27 cells were seeded in 6-well plates at 1×10^3 cells per well. Cells were maintained in complete medium for 10 days. Cells were then washed twice with PBS, fixed with methanol and stained with 0.1% crystal violet. Colonies were counted under a microscope.

Apoptosis assay: For apoptosis analysis, cells were stained with Annexin V-FITC and propidium iodide (PI) (Genecopoeia) according to the manufacturer's instructions. Briefly, SCC25 and CAL27 cells were trypsinized, centrifuged and wash twice with cold PBS. Cells were re-suspended in binding buffer containing Annexin V-FITC and PI. After 15 min of incubation in the dark, cells were analyzed by flow cytometry.

Wound healing assay: Confluent monolayers of cells were cultured in 6-well plates. Wounds were scratched in the monolayer with a 200 μl plastic pipette tip. The culture medium was replaced with serum-free medium. The migration of cells into the wounded area was photographed under an inverted phase contrast microscope (Olympus) at 0 h and 48 h time points. Three different wound sites in each group were photographed, and the migratory activity of cells were evaluated/the relative cell motility was calculated.

Transwell assay: Transwell assay was performed using matrigel-coated transwell chambers (Corning) containing 8 μm pores. Cells (5×10^4 cells per well) suspended in serum-free medium were added to the upper chambers. DMEM supplemented with 10% FBS was added to the lower chambers as chemoattractant. Following incubation at 37 °C with 5% CO_2 for 24 h, the non-invading cells on the upper surface of matrigel membrane were scraped off with a cotton swab. Cells migrating through the membrane to the lower surface were fixed with 4% paraformaldehyde and stained with DAPI (Servicebio). The stained cells were photographed under a fluorescence microscope (Olympus).

TUNEL assay: Apoptotic DNA fragmentation was detected using the terminal deoxynucleotidyl transferase-mediated dUTP nick end-labeling (TUNEL) kit (Roche) according to the manufacturer's instructions. Briefly, SCC25 and CAL27 cells were seeded in 48-well plates at 3×10^4 cells per well. Cells were fixed with 4% paraformaldehyde and then permeabilized in 0.1% Triton X-100. After

washing twice with PBS, cells were incubated with TUNEL reaction mixture for 1 h at 37 °C in a humidified atmosphere. Cells were then treated with DAPI for nuclear staining. TUNEL-positive cells were counted under a fluorescence microscope (Olympus).

Intracellular Reactive Oxygen Species (ROS) measurement: The level of intracellular ROS was examined using reactive oxygen species assay kit (Beyotime). Briefly, SCC25 and CAL27 cells were seeded in 48-well plates at 3×10^4 cells per well. Cells were incubated with 10 µM DCFH-DA for 20 min at 37 °C. DCFH-DA is oxidized by ROS to its fluorescent product DCF in viable cells. Cells were washed three time with PBS and treated with Hochest 33342 (Genecopoia) for nuclear staining. The fluorescence intensity of DCF was examined under a fluorescence microscope (Olympus).

Nucleus-cytoplasm fractionation: Nuclear and cytoplasmic fractions were isolated from SCC25 and CAL27 cells using the PARIS kit (Ambion) according to the manufacturer's instructions. Briefly, cells were washed once with PBS and resuspended with ice-cold fractionation buffer. Cells were centrifuged at 500 × *g* for 5 min, and the supernatant containing the cytoplasmic fraction was aspirated off. The nuclear pellet was lysed with disruption buffer. RNA is harvest by adding ethanol and drawing through a filter cartridge. Nuclear and cytoplasmic RNA was converted to cDNA and analyzed by qPCR.

RNA fluorescent in situ hybridazation (RNA FISH): The subcellular localization of *HITTERS* was examined by RNA FISH. The sequence of FITC-labeled FISH probe (Genepharma) is listed in supplementary table 2. Cells were fixed in 4% paraformaldehyde for 15 min, permeabilized with 0.1% Triton X-100, and incubated with 2 × SSC buffer. Cells were incubated with FISH probe in Hybridization buffer (Genepharma) in the dark at 37 °C overnight, washed twice with 2 × SSC buffer, and treated with DAPI for nuclear staining. Images were captured by a fluorescence

microscope (Olympus).

Rapid amplification of cDNA ends (5'- and 3'-RACE): RNA was extracted from SCC25 cells using the Trizol reagent (Invitrogen). Total RNA was polyadenylated by treating with poly(A) polymerase buffer and 0.5 mM ATP. First-strand cDNA was synthesized from poly(A)$^+$ RNA with an adaptor primer and a RevertAid First Strand cDNA Synthesis Kit (Thermo Scientific). A poly(G) tail was added to the 3' end of the cDNA using TdT and dGTP. The dG-tailed cDNA underwent two rounds of PCR amplification including nested PCR to increase the specificity. PCR products were separated by 1% agarose gel and individual bands were excised for sequencing. The sequence of primers is listed in supplementary Table 1.

RNA extraction and qPCR: Total RNA was extracted using RNAiso Plus (Takara) added with Acryl Carrier (Solarbio). First-strand cDNA was synthesized using RevertAid RT kit (Thermo Fisher Scientific). qPCR was carried out using gene-specific primers, cDNA, SYBR Green qPCR Master Mix, and an ABI Q6 Flex platform (Thermo Fisher Scientific). The primers for each gene are listed in supplementary Table 2.

Western blotting: Proteins were extracted by RIPA buffer (Beyotime) and separated by 10% SDS-PAGE (Bio-Rad). Proteins were transferred to 0.22 μm PVDF membranes (Millipore). Membranes were blocked with 5% BSA in TBST containing 0.1% Tween-20, probed with primary antibodies (Supplementary table 3). Blots were incubated with HRP-conjugated secondary antibodies, and detected with ECL reagent (Millipore).

Co-immunoprecipitation (IP), RNA IP (RIP) and MS2bs-MS2bp RNA pull-down: For co-IP, cells were lysed by IP buffer (Beyotime), incubated with anti-Rad50, anti-MRE11 or anti-NBS1 antibody overnight at 4 °C. Protein A/G magnetic beads (Bimake) were subsequently added and the mixture were incubated at 4 °C for 1 h. The beads were collected by magnetic separation and the

non-binding supernatant was discarded. Bound protein was eluted by boiling at 100 °C for 5 min. Samples were subjected to western blotting to evaluate the interaction of Rad50, Mre11 and NBS1. For RIP, the Imprint RIP Kit (Sigma) was used following the instruction of the manufacture to determine the interaction between *HITTERS* and RAD50/MRE11. For MS2bs-MS2bp RNA pull-down, cells were co-transfected with pMS2-GFP (Addgene) and pcDNA3.1(+)-12×MS2bs empty/ HITTERS truncations vector. The pcDNA3.1(+)-12×MS2bs was generated via cloning the 12×MS2bs sequence from pSL-MS2-12X (Addgene) and inserting it into pcDNA3.1(+). After 48h, the co-transfected cells were harvested and lysed. After incubation with anti-GFP antibody overnight at 4 °C, the captured RNA-protein complex was captured by protein A/G magnetic beads (Bimake). Proteins were eluted by boiling and analyzed via wester blotting. The antibodies for co-IP, RIP and MS2bs-MS2bp RNA pull-down are listed in supplementary table 3.

Supplementary Figure Capture

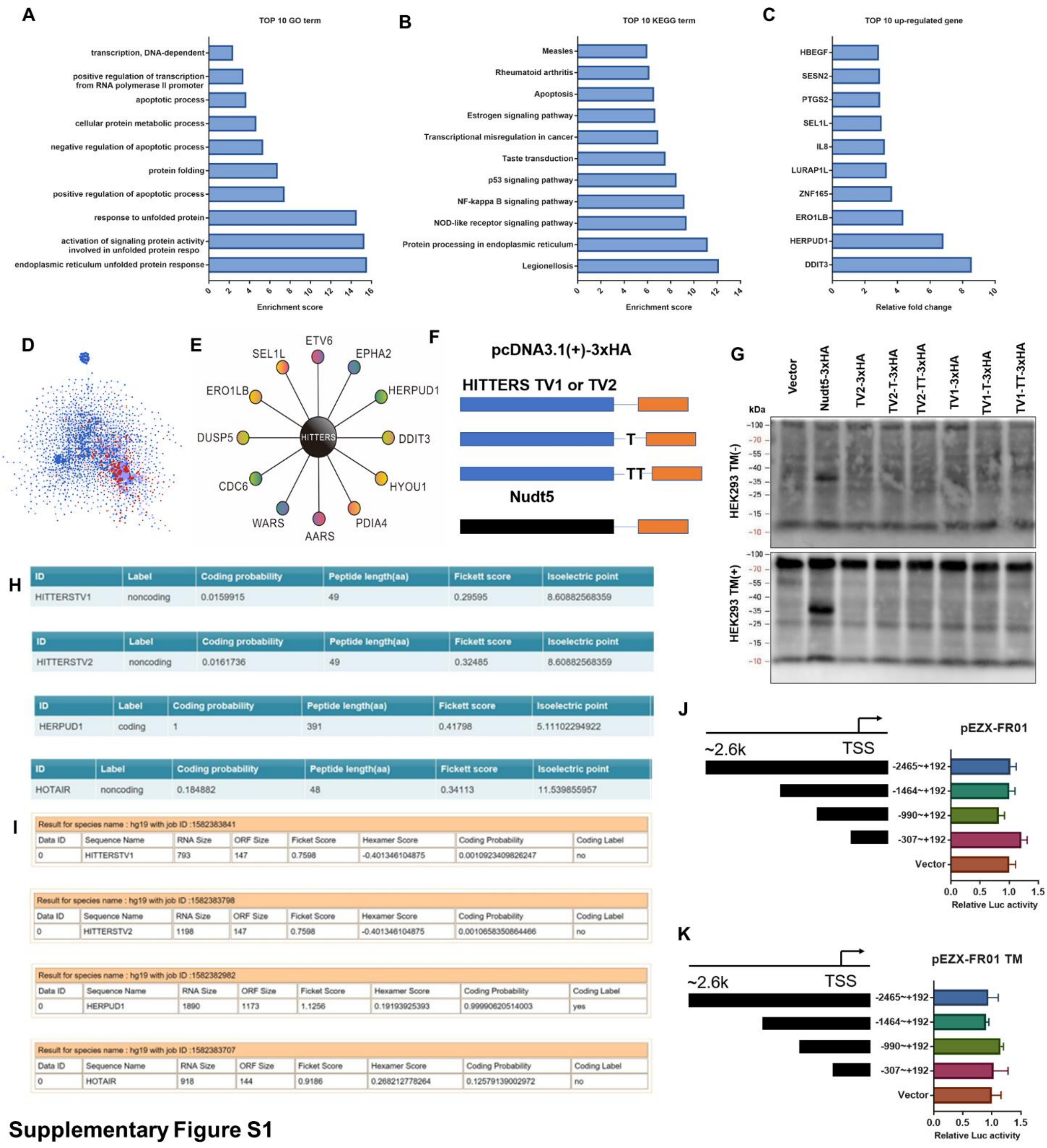

Supplementary Figure S1

A-C, Top 10 GO term (A), top 10 KEGG pathway (B) and top 10 most-significantly up-regulated gene (C) of HTA 2.0 mRNA microarray for gene expression profiles of non-treated and TM-treated SCC25 cells. **D and E,** lncRNA-mRNA co expression network of HTA 2.0 lncRNA and mRNA microarray. An extensive co-expression pattern between lncRNA and mRNA could be observed (D),

and *HITTERS* was highly correlated (R>0.99) with multiple ER stress-related genes (E). **F,** The schematic representation of 3xHA tagged *HITTERS* exploring the coding potential. **G,** Western blot indicated *HITTERS* lacked coding capability, no matter whether ER stress was triggered or not. HEK293 cells were transfected with recombined plasmid for 48h before harvesting. **H and I,** Online bioinformatic tools CPC (H) and CPAT (I) both predicted that similar to *HOTAIR*, two *HITTERS* TVs had no protein coding ability. In contrast, *HERPUD1* had strong coding potential. CPC, Coding Potential Calculator (http://cpc2.cbi.pku.edu.cn/). CPAT, Coding Potential Assessment Tool (http://lilab.research.bcm.edu/cpat/). **J and K,** Dual-luciferase reporter assay using pEZX-FR01 showed the potential promoter DNA fragments of *HITTERS* lacked transcription activity no matter threated with TM (10 μg/ml, 6h) or not. One-way ANOVA and Dunnett-t test were used, “Vector” was the control, all differences were none significant.

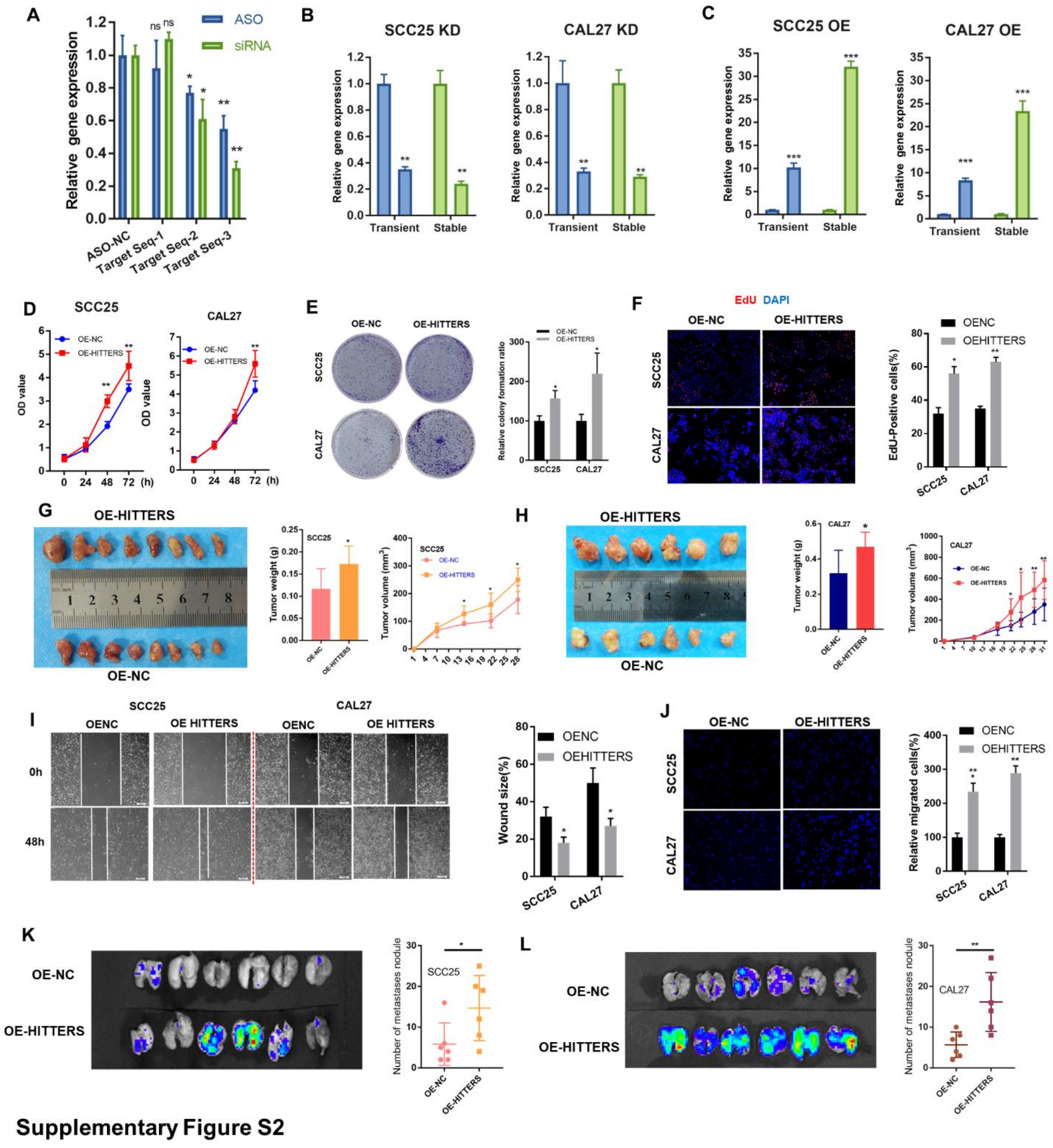

Supplementary Figure S2

A, qPCR results on the knock-down efficiency of ASO and si-RNA. Target 3 sequence was selected for further experiment. SCC25 cells were transfected with ASO or siRNA for 48h before harvesting. One-way ANOVA and Dunnett-t test were used for ASO or siRNA, respectively. **B and C**, qPCR results on the transient or stable knock-down/overexpression efficiency. Student t test was used. **D to F**, CCK8 (A), colony formation test (B) and EdU incorporation test (C) confirmed that

over-expression of *HITTERS* promoted OSCC proliferation in vitro. **G and H**, Stably over-expression of *HITTERS* significantly promoted tumor volume and tumor weight in SCC25 (D) and CAL27 (E) subcutaneous xenograft model. **I and J**, Wound healing test (F) and transwell assay (G) confirmed over-expression of *HITTERS* prromoted OSCC migration and invasion ability in vitro. **K and L**, Stably over-expression of *HITTERS* significantly promoted SCC25 (H) and CAL27 (I) pulmonary metastasis nodule formation ability in vivo. The GFP fluoresces imaging of lungs was also presented. For **D to L**, Student t test was used. For **D, G and H**, Student t test was used for each time point. Note: *, P< 0.05; **, P < 0.01, ***, P < 0.001.

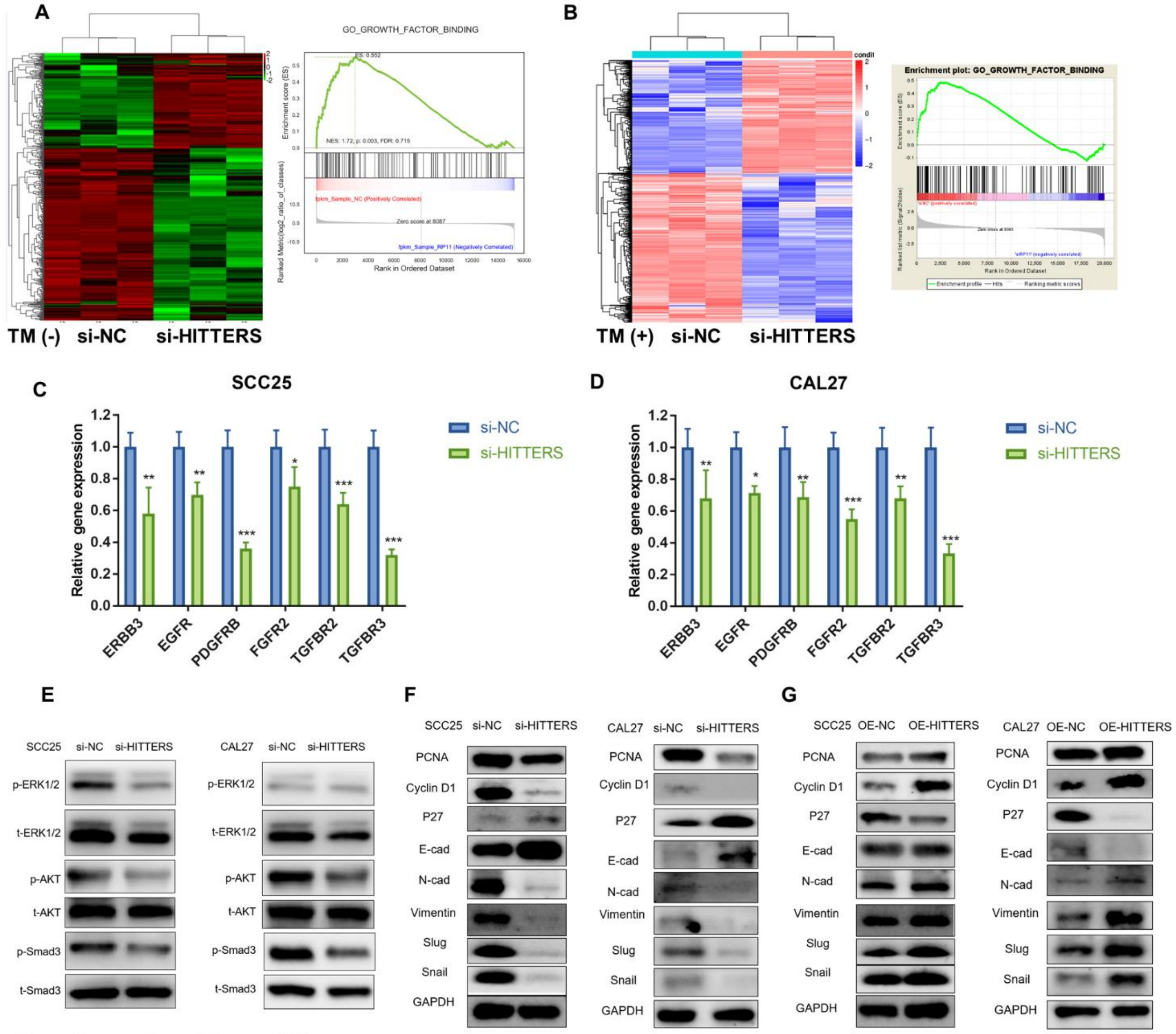

Supplementary Figure S3

Supplementary Figure S3

Receptor tyrosine kinases and TGF-β receptors are downstream targets of HITTERS. **A and B,** Heat map and GSEA analysis of mRNA-sequencing for gene expression profiles change after *HITTERS* knock-down. SCC25 cells were transfected with siRNA for 48h and treated with (B) or without (A) TM (10 μg/ml, 12h). **C and D,** qPCR results on RTKs and TGFβR change after *HITTERS* knock-down in SCC25 (C) and CAL27 (D) cells. The Student t test was used. **E,** Western blot showed knock-down of *HITTERS* significantly decreased phosphorylation of AKT, ERK1/2 and SMAD3. **F and G,** western blot showed the cell proliferation and EMT were regulated by *HITTERS* knock-down (F) or overexpression (G). Note: *, P< 0.05; **, P < 0.01; ***, P < 0.001.

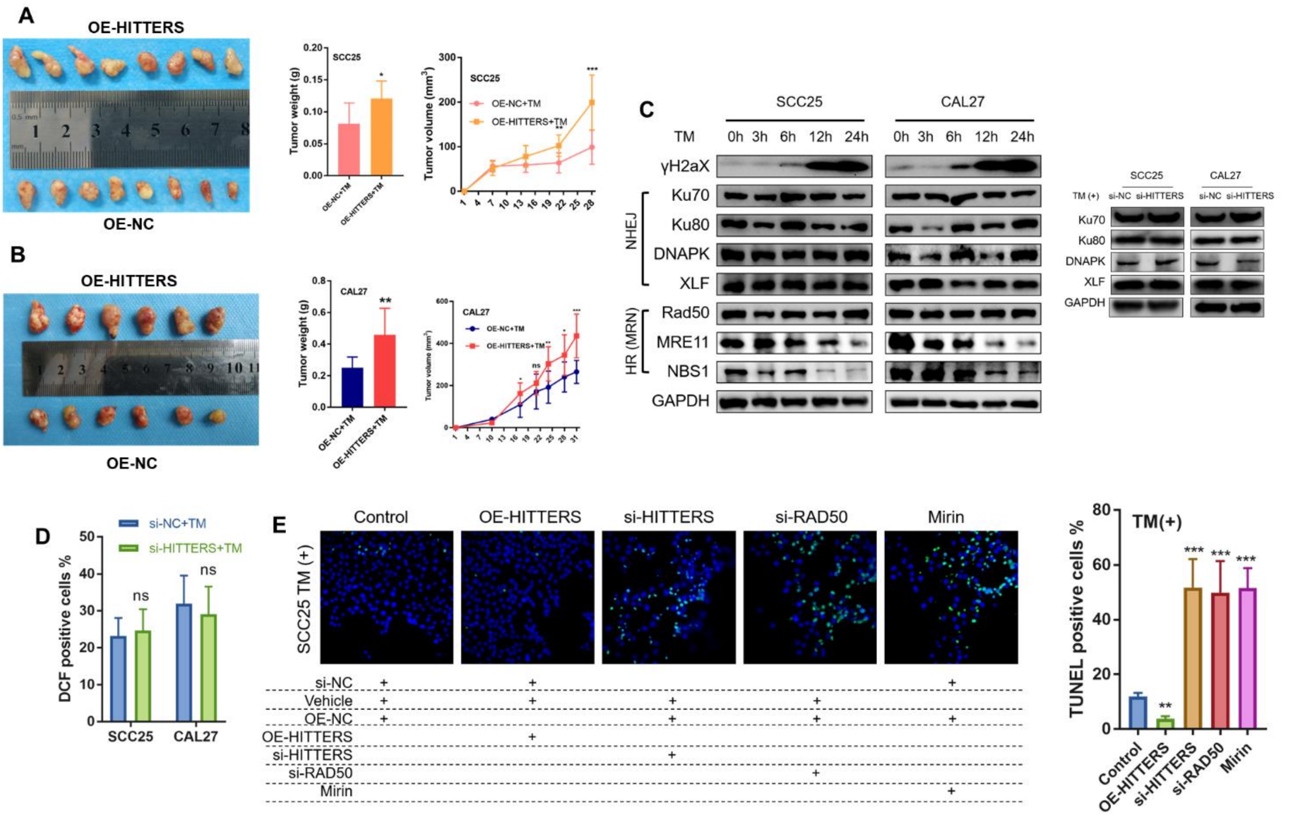

Supplementary Figure S4

Supplementary Figure S4

A and B, Stably over-expression of *HITTERS* causing SCC25 (A) and CAL27 (B) more resistance to ER stress in vivo, reflected by a significantly promotion of tumor volume and tumor weight in subcutaneous xenograft model. All BALB/c nude mice were intraperitoneally injected with TM, twice a week, after tumor bearing. Student t test was used. **C,** Induction of ER stress by TM (10 μg/ml) significantly promoted the level of DNA damage marker γ-H2AX and suppressed HR related proteins in a time-dependent manner, however, NHEJ related proteins did not change significantly. Knocking-down *HITTERS* had no influence on the expression of NHEJ related proteins. Cells were transfected with siRNA or plasmid for 48h and then treated with TM (10 μg/ml) for 24h.**D,** DCF staining indicated knock-down of *HITTERS* did not influence ROS production under ER stress. Cells were transfected with siRNA for 48h and treated with TM (10 μg/ml, 24h). **E,** TUNEL assay on SCC25 cells found knock-down of *HITTERS* or inhibiting MRN complex

promoted DNA damage, whereas overexpression of *HITTERS* suppressed DNA damage. For C and D, cells were transfected with siRNA or plasmid for 48h and then treated with TM (10 μg/ml) or Mirin (100μM) for 24h. Note: ns, no significance; P< 0.05; **, P < 0.01; ***, P < 0.001.

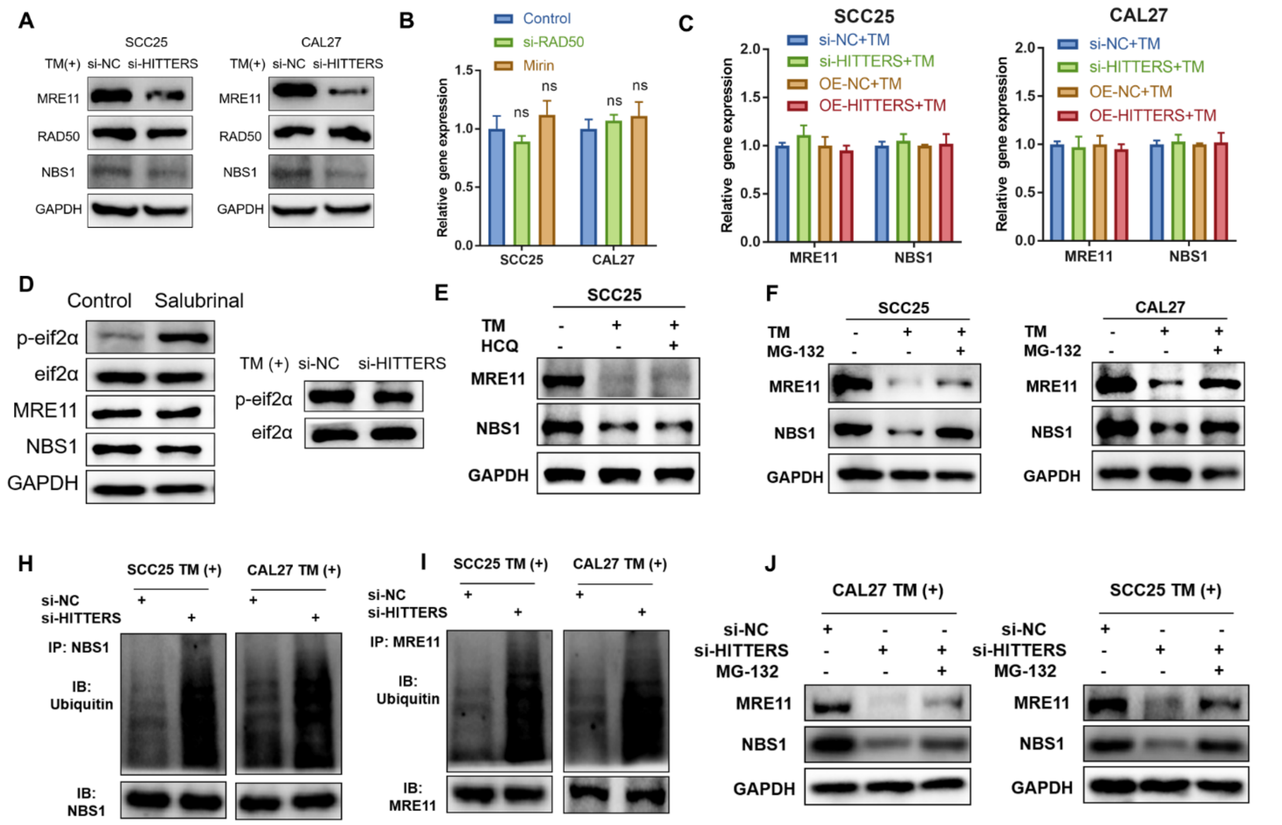

Supplementary Figure S5

Supplementary Figure S5

A, Western blot results showed under ER stress, knock-down of *HITTERS* significantly decreased the protein level of MRE11 and NBS1, but had no effect on RAD50 expression. Cells were transfected with siRNA for 48h and then treated with TM (10 μg/ml) for 12h. **B,** qPCR results indicated inhibiting MRN complex did not affect *HITTERS* expression under ER stress. Cells were treated with TM (10 μg/ml) for 6h. One-way ANOVA and Dunnett-t test were used. ns, no significance. **C,** qPCR results indicated *HITTERS* had no influence on the RNA level of MRE11 and NBS1. Cells were transfected with siRNA for 48h and then treated with TM (10 μg/ml) for 12h.

The Student t test was used, and all were non-significant. **D,** Treat SCC25 cells with Salubrinal (50μM, 24h) significantly promoted eif2a phosphorylation, but had no impact on MRE11 and NBS1 protein level. Also, knock-down of *HITTERS* had no impact on eif2a phosphorylation (cells were transfected with siRNA for 48h and then treated with TM for 3h). **E and F,** ER stress could significantly decrease the protein level of NBS1 and MRE11, and inhibiting authophagy system via HCQ could not rescue the degradation (E), whereas inhibiting proteasome via MG-132 could rescue this degradation (F). For E, SCC25 cells were treated with HCQ for 24h, and then treated with HCQ and TM for another 24h. For F, cells were treated TM for 12h, and then treated with MG-132 and TM for another 12h. **H and I,** IP found that knock-down of *HITTERS* significantly increased the ubiquitination level of NBS1 (H) and MRE11 (I) in both SCC25 and CAL27 cells. Cells were transfected with siRNA for 48h and then treated with TM (10 μg/ml) for 12h. **J,** Inhibiting proteasome could rescue the degradation of NBS1 and MRE11 caused by *HITTERS* depletion. Cells were transfected with siRNA for 48h and then treated with TM (10 μg/ml) for 24h.

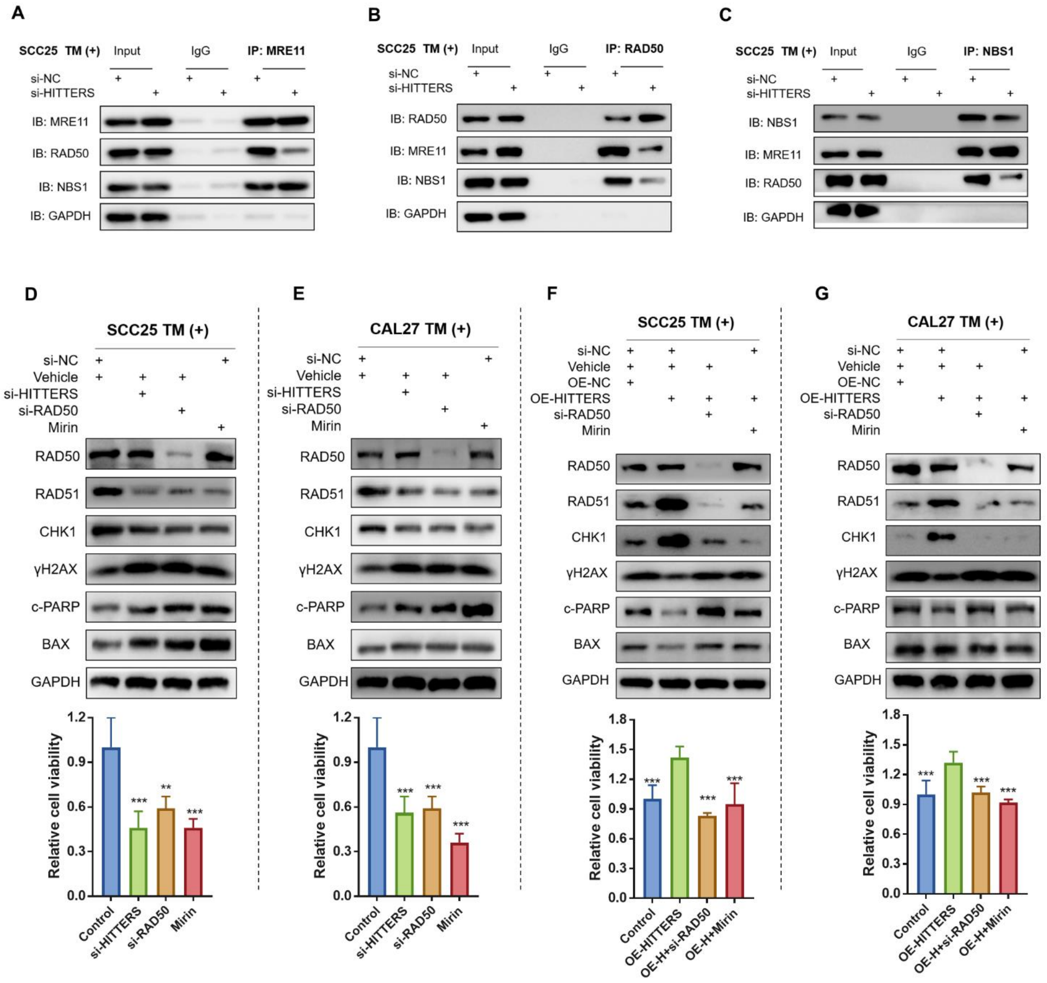

Supplementary Figure S6

Supplementary Figure S6

A to C, co-IP results showed the interaction between MRE11 and RAD50 in SCC25 cells were reduced after *HITTERS* knock-down. The interaction between MRE11 and NBS1 remained no change after *HITTERS* knock-down. Cells were transfected with siRNA for 48h and then treated with TM (10 μg/ml) for 6h. **D and E,** Inhibiting MRN complex by RAD50 knock-down or small molecule inhibitor Mirin had the same impact on SCC25 and CAL27 cells as *HITTERS* knock down, that is, promoting the expression of apoptosis and DNA damage marker, and suppressing DNA repair protein and cell viability (measured by CCK8). One-way ANOVA test and Dunnett-t test were used was used for cell viability; “Control” was selected as standard. **F and G,** Inhibiting MRN complex by *RAD50* knock-down or Mirin abolished the protective effect of *HITTERS*

over-expression. "OE-H" stands for "OE-*HITTERS*". One-way ANOVA test and Dunnett-t test were used was used for cell viability; "OE-H" was selected as standard. For **D to G**, cells were transfected with siRNA or plasmid for 48h and then treated with TM (10 μg/ml) or Mirin (100μM) for 24h. Note: **, P < 0.01; ***, P < 0.001.

Supplementary table 1 Primer sequence for qPCR and RACE, and oligos for CRISPR

Application	Note	Forward Sequence 5'-3'	Reverse Sequence 5'-3'
**qPCR**	HITTERS	CAGCTGCCTTCCAGGGTAAA	CTTCGGGCCACAGCTAAAAC
	HERPUD1	CCGGTTACACACCCTATGGG	TGAGGAGCAGCATTCTGATTG
	BIP	TTCTTGTTGGTGGCTCGACT	GTCAGCATCTTGGTGGCTTT
	CHOP	GGAAACAGAGTGGTCATTCCC	CTGCTTGAGCCGTTCATTCTC
	XBP1s	CCGCAGCAGGTGCAGG	GGGGCTTGGTATATATGTGG
	ATF4	CCTTCACCTTCTTACAACCT	GTAGTCTGGCTTCCTATCTC
	ATF6	GCCGCCGTCCCAGATATTA	CCAGCCTGTGAAAGAGTCCC
	PERK	CTCAGCGACGCGAGTACC	CGGTCGCAACTCTGTCTCAT
	GAPDH	AGGTCCACCACTGACACGTT	GCCTCAAGATCATCAGCAAT
	U6	CTCGCTTCGGCAGCACA	AACGCTTCACGAATTTGCGT
	GAS5	CCATGGATGACTTGCTTGGG	TGCATGCTTGCTTGTTGTGG
	MALAT1	GGATCCTAGACCAGCATGCC	AAAGGTTACCATAAGTAAGTTCCAGAA
	ERBB3	GACCCAGGTCTACGATGGGAA	GTGAGCTGAGTCAAGCGGAG
	EGFR	AGGCACGAGTAACAAGCTCAC	ATGAGGACATAACCAGCCACC
	PDGFRB	AGCACCTTCGTTCTGACCTG	TATTCTCCCGTGTCTAGCCCA
	FGFR2	GGAAAGTGTGGTCCCATCTGA	TCCAGGTGGTACGTGTGATTG
	TGFBR2	GTAGCTCTGATGAGTGCAATGAC	CAGATATGGCAACTCCCAGTG
	TGFBR3	GGAAAACACCGAGTCGGAATAC	GCGGAAAACCTTGGAGGTAAT
	NBS1	ATGGAACAGTGAGGAATGGAG	TTGGAAGGTGAGAGTGATGTAG
	MRE11	ATGAAGTCCGTGAGGCTATG	GGTTGCTGCTGAGATGCTA
	Primer 1 (Figure 2A)	GAGTCTCGCTCTGTCATTCA	CAGTGGTGCGATCTTGGCTC
	Primer 2 (Figure 2A)	CAGCTGCCTTCCAGGGTAAA	CTTCGGGCCACAGCTAAAAC
	Primer 3 (Figure 2A)	AACACAGTCCCTGCCTTCAA	AGCACCCTGATAAGCTCACTG
	Primer 1 (Figure 7B)	GGTGACAGAGCAAGACCCT	GTGGTTTGTGAGCCTGGAAG
	Primer 2 (Figure 7B)	TGGCTTGGTTGTATGGTCCC	GAAGGCAGCTGTGGTCTGAT
	Primer 3 (Figure 7B)	CACTGACTGTTAGCATGGCC	TCCCCTAGATGTGACAACCA
**5' RACE**	Nested PCR primer 1	GGCCACGCGTCGACTAGTAC CCCCCCCCCCCCC	ATGTCAGGTATGTGAAGGTTCCC
	Nested PCR primer 2	GGCCACGCGTCGACTAGTAC	TCAGGGCCATGCTAACAGTCAG
**3' RACE**	Nested PCR primer 1	CCTTCCAGGCTCACAAACCAC	CGAAAGCGACAAGGCCGTGATCCCGAAAGC TTTTTTTTTTTTTTTTTTTTTTTTTTVN
	Nested PCR primer 2	CTGAGGCATGGAACAGAAAAC	CGAAAGCGACAAGGCCGTGATCCCGAAAGC
**CRISPR**	sgDNA set-1F	CACCGCCCCACGTGGAAACCTACG	AAACCGTAGGTTTCCACGTGGGGC
	sgDNA set-1R	CACCGTTCCCCGTAGGTTTCCACGT	AAACACGTGGAAACCTACGGGGAAC
	sgDNA set-2F	CACCGGGCGCGGGGCTTCTATAAA	AAACTTTATAGAAGCCCCGCGCCC
	sgDNA set-2R	CACCGTATAAAAGGCGCCCGAAGCG	AAACCGCTTCGGGCGCCTTTTATAC

Sequencing primer	CCACTTCAGGAATCGGAGAA	CTTGGAGGGTGAAATGACTC

Supplementary table 2 Nucleotide sequence for ChIRP, siRNA and FISH

**Application**	**Note**	**Sequence 5'-3'**
**ChIRP**	Antisense Probe 1	GGACCATACAACCAAGCC
	Antisense Probe 2	GCCTCAGTATGGCTACAT
	Antisense Probe 3	CTTTACCCTGGAAGGCAG
	Antisense Probe 4	GGGCCACAGCTAAAACCA
	Antisense Probe 5	ACGAAGACCCATCACTCT
	Antisense Probe 6	GCGGGGAGAATGCATATA
	Antisense Probe 7	CCCATCTACAGAGGCATT
	Antisense Probe 8	GTCCATCTTAGCTGATGT
**siRNA target**	HITTERS 1	GGACAACUCCCACAGUAAA
	HITTERS 2	GCAAGGGAACCUUCACAUA
	**HITTERS 3**	**GCUCACAAACCACAAGUAU**
	ATF4	CUGCUUACGUUGCCAUGAU
	ATF6	GCAUCUGCCUGGUUACUUA
	XBP1s	UGCCAAUGAACUCUUUCCC
	HERPUD1	GGCUUGUCUUCAAGACUUUC
	RAD50	GGAGAAGGAAAUACCAGAA
	NC	UUCUCCGAACGUGUCACGU
**FISH**	Probe 1	CTGTGGTCTGACAAAGGCAA
	Probe 2	CTGACTTTACCCTGGAAGC
	Probe 3	TTTCAGGGCCATGCTAACG

Supplementary table 3 Antibody list

Application	Target	Dilution	Manufacturer	Catalog
**Western blot**	ATF6	1:500	CST	65880
	p-eIF2a	1:500	CST	3398
	eIF2a	1:500	CST	5324
	ATF4	1:1000	CST	11815
	BIP	1:2000	Huabio, China	ER40402
	GAPDH	1:1000	CST	5174
	c-PARP	1:1000	CST	5625
	BAX	1:1000	CST	5023
	CytoC	1:1000	CST	11940
	HERPUD1	1:3000	Abcam	Ab150424
	γH2AX	1:2000	Huabio, China	ET1602-2
	MER11	1:1000	CST	4847
	RAD50	1:2000	Genetex	GTX70228
	NBS1	1:500	CST	14956
	ATM	1:1000	CST	2873
	ATR	1:1000	Abcam	Ab2905
	CHK1	1:500	CST	2360
	CHK2	1:500	CST	6334
	RAD51	1:1000	Abcam	Ab63801
	TP53	1:2000	SCBT	SC-126
	Ubiquitin	1:2000	Abcam	Ab7780
	GFP	1:2000	Abcam	Ab290
	DNAPK	1:1000	Huabio, China	ET1610-12
	Ku70	1:500	Huabio, China	ET1703-95
	Ku80	1:1000	Huabio, China	ET1610-40
	XLF	1:5000	Abcam	ab189917
	HA tag	1:2000	CST	3724
	p-ERK1/2	1:2000	CST	9101
	t-ERK	1:2000	CST	4695
	p-AKT	1:1000	CST	4060
	t-AKT	1:1000	CST	4691
	p-Smad3	1:1000	CST	9520
	t-Smad3	1:1000	CST	9523
	PCNA	1:1000	CST	13110
	CyclinD1	1:1000	CST	2978
	P27	1:1000	CST	3686
	E-cad	1:1000	CST	3195
	N-cad	1:1000	CST	13116
	Vimentin	1:1000	CST	5741
	Slug	1:1000	CST	9585
	Snail	1:1000	CST	3879
**RIP and IP**	MRE11	5μg per reaction	CST	4847
	RAD50	5μg per reaction	Genetex	GTX70228
	NBS1	5μg per reaction	CST	14956

	GFP	2μl per reaction	Abcam	Ab290

Supplementary table 4 Nucleotide sequence for HITTERS

>HITTERS TV1

GGGTGACAGAGCAAGACCCTGTCTTAGAATAAATACATAATAAATAGTATGTATTCTGGC
ACTTTCGATACAAGGAATTCATGGCTTGGTTGTATGGTCCCAAGAACATATCAATCCTGT
GTTAATATAAGAATATTATCTTGTCCTCTAGATAAGCTACCTTACCTTCCAGGCTCACAA
ACCACAAGTATGTAGCCATACTGAGGCATGGAACAGAAAACTGTATTTGTTTTGAATGAG
AACACATTTGCCTTTATCAGACCACAGCTGCCTTCCAGGGTAAAGTCAGGCACTGACTGT
TAGCATGGCCCTGAAAGGCAAGGGAACCTTCACATACCTGACATTTGGTTTTAGCTGTGG
CCCGAAGCAGTAGTTCTCAACTGGGGGTGGTTTTGTACCCTCTCTCCCCAGGGGACATTT
GGCAATGTGTAGATATTTTTGGTTGTCACATCTAGGGGAAGTGGTCCTGCTGGCGTCTAG
TTAGTAGAAGCAAGAGTGCTGCTGAATTTTCTACAATGCACAGGACAACTCCCACAGTAA
ATATTTGGCTCAATATGTCACTAGTGCCAAAGCTGAGAAAGCCTGGCCTAGAGTGATGGG
TCTTCGTGGTTGAAACTAAAAGAAGAAATTTTCCTGTATAGTAAAAATGGATTTTTATTT
TAGCTTTTAAAAATAAAAAAGGAATAATTAGAATATAATGAACACTCAAATATCCACAAT
TAGTCATTGTTAATATTTTATTATATTTAAGACTTGGGATTTAATTGGTTTCTTCAAATA
AAACGTTTAGTTA

>HITTERS TV2

GGGTGACAGAGCAAGACCCTGTCTTAGAATAAATACATAATAAATAGTATGTATTCTGGC
ACTTTCGATACAAGGAATTCATGGCTTGGTTGTATGGTCCCAAGAACATATCAATCCTGT
GTTAATATAAGAATATTATCTTGTCCTCTAGATAAGCTACCTTACCTTCCAGGCTCACAA
ACCACAAGTATGTAGCCATACTGAGGCATGGAACAGAAAACTGTATTTGTTTTGAATGAG
AACACATTTGCCTTTATCAGACCACAGCTGCCTTCCAGGGTAAAGTCAGGCACTGACTGT
TAGCATGGCCCTGAAAGGCAAGGGAACCTTCACATACCTGACATTTGGTTTTAGCTGTGG
CCCGAAGCAGTAGTTCTCAACTGGGGGTGGTTTTGTACCCTCTCTCCCCAGGGGACATTT
GGCAATGTGTAGATATTTTTGGTTGTCACATCTAGGGGAAGTGGTCCTGCTGGCGTCTAG
TTAGTAGAAGCAAGAGTGCTGCTGAATTTTCTACAATGCACAGGACAACTCCCACAGTAA
ATATTTGGCTCAATATGTCACTAGTGCCAAAGCTGAGAAAGCCTGGCCTAGAGTGATGGG
TCTTCGTGGTTGAAACTAAAAGAAGAAATTTTCCTGTATAGTAAAAATGGATTTTTATTT
TAGCTTTTAAAAATAAAAAAGGAATAATTAGAATATAATGAACACTCAAATATCCACAAT
TAGTCATTGTTAATATTTTATTATATTTAAGACTTGGGATTTAATTGGTTTCTTCAAATA
AAACGTTTAGTTAATATTTTCTGTAGTCTCTAGAATCATATATTTAAAAGACCAAATGTA
GGTTGTCATTATGTTATATATAATTATAGCTTTGTAGGTATATGCATTCTCCCCGCTCTG
TTCAGGTTTTCAAGGGTAAGGTTTAGTTCGTCATAAGCATTTATTGAGTGCATACTATGT
GCCAGGTGCTGTTTATATGGCTGGGTGTGCAATGATGAATAAAACACAGTCCCTGCCTTC
AAGGACCTTACAGACTGGTGAGAATGTCTGGGAGACAGTGTGATCAAATGCCTCTGTAGA
TGGGTCTTGCAGTGAGCTTATCAGGGTGCTGCTGTGATTAGAGGTGGGGAGCCTTGGATT
CTTGAGTAAGTCTCACTGTGACATCAGCTAAGATGGACTTTTATGTGCTTCCTTTGAA

Supplementary table 5 Correlation between *HITTERS* level and OSCC clinicopathological features

Characteristics	*HITTERS*		*P* value
	**Low expression**	**High expression**	
Gender			
Male	14	16	0.551
Female	10	8	
Age(years)			
≤59	15	11	0.193
＞59	9	13	
Pathogenic site			
tongue	12	19	0.034
others	12	5	
Smoke			
no	11	10	0.500
yes	13	14	
Alcohol drinking			
no	11	11	0.614
yes	13	13	
T stage of tumor			
I-II	15	6	**0.008**
III-IV	9	18	
N stage of tumor			
N0	21	12	**0.006**
N1-2	3	12	
Clinical stage			
I-II	15	5	**0.004**
III-IV	9	19	
Pathological grade			
I	14	17	0.273
II-III	10	7	

Note: A total of 48 OSCC were included. HITTERS expression was divided by medium split (High=24, low=24). Analyzed by Chi-square test.
